# Investigation of the Proton-Bound Dimer of Dihydrogen Phosphate and Formate Using Infrared Spectroscopy in Helium Droplets

**DOI:** 10.1021/acs.jpca.4c01632

**Published:** 2024-05-21

**Authors:** América Y. Torres-Boy, Martín I. Taccone, Carla Kirschbaum, Katja Ober, Tamar Stein, Gerard Meijer, Gert von Helden

**Affiliations:** † Fritz Haber Institute of the Max Planck Society, 14195 Berlin, Germany; ‡ Institute of Chemistry and Biochemistry, Freie Universität Berlin, 14195 Berlin, Germany; § Institute of Chemistry and Fritz Haber Center for Molecular Dynamics, Hebrew University of Jerusalem, 91904 Jerusalem, Israel

## Abstract

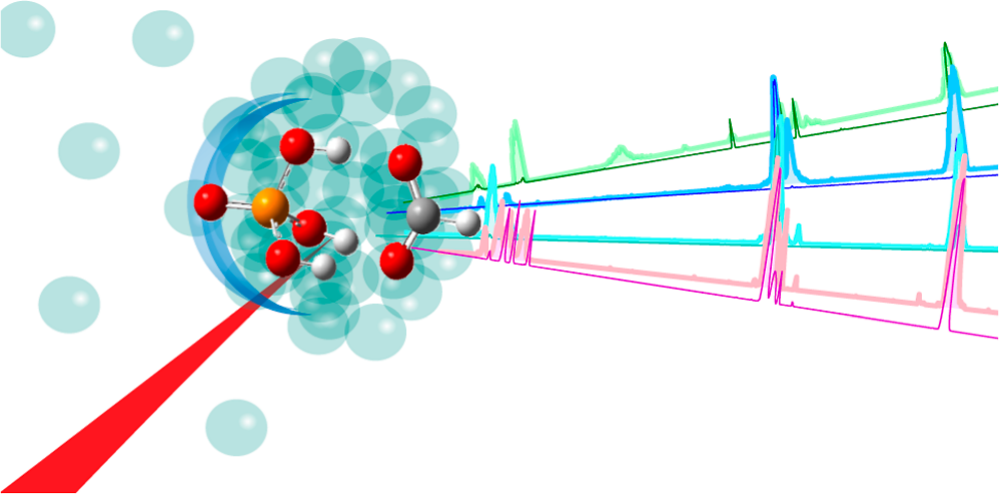

Understanding the structural and dynamic properties of proton-bound complexes is crucial for elucidating fundamental aspects of chemical reactivity and molecular interactions. In this work, the proton-bound complex between dihydrogen phosphate and formate, and its deuterated counterparts, is investigated using IR action spectroscopy in helium droplets. Contrary to the initial expectation that the stronger phosphoric acid would donate a proton to formate, both experiment and theory show that all exchangeable protons are located in the phosphate moiety. The experimental spectra show good agreement with both scaled harmonic and VPT2 anharmonic calculations, indicating that anharmonic effects are small. Some H-bending modes of the nondeuterated complex are found to be sensitive to the helium environment. In the case of the partially deuterated complexes, the experiments indicate that internal dynamics leads to isomeric interconversion upon IR excitation.

## Introduction

Phosphate-containing molecules come in diverse sizes and with different functionalities. They are ubiquitous in nature and play key roles in numerous chemical processes. In inorganic chemistry, orthophosphoric acid (H_3_PO_4_) has been shown to play a fundamental role in prebiotic chemistry,^1−3^ as well as in complex atmospheric processes.^4,5^ Further, due to its high proton conductivity, it is widely used as a proton carrier in the development of ionic-liquids membrane fuel cells.^6−9^

Of particular importance are the many biochemical processes where phosphate-containing biomolecules take part, and several comprehensive reviews on the biophysics and biochemistry of phosphorus have been published.^10−12^ Those processes include protein synthesis,^10,13^ metabolism,^14^ signaling,^15^ energy production,^16^ and phosphoryl transfer.^17,18^ In addition, phosphate complexation motifs^19,20^ provide strong intra- and intermolecular interactions and are vital for structure formation in, for example, DNA and RNA, phosphorylated proteins, carbohydrates, and lipids. Often, acid–base interactions are involved in which protons participate in hydrogen bonding and can be localized, shared, or transferred. In order to better understand hydrogen bonding and acid–base chemistry involving phosphate units, phosphoric acid neutral and ionic clusters have been extensively investigated.^21−23^ Such studies on size-selected and isolated systems can provide detailed insights into the structure and energetics of well-defined systems, and the interactions within clusters can serve as models related to, for example, noncovalent bonding found in biomolecules^22−24^ or phosphate–water interactions.^21^

An acid–base interaction might involve the transfer of protons from the acid to the base. Whether this process takes place depends on the properties of the acid and the base as well as on their environment. When considering isolated noninteracting molecules, the energetics is given by the relative proton affinity (PA) of the involved species. For systems in aqueous solution where interactions with the surroundings are important, the p*K*_a_ values give a measure for the acid–base chemistry. However, when the acid and base molecules are directly interacting, and/or not in aqueous solutions, PA and p*K*_a_ values do not allow us to predict where the proton will be located. The location of a proton in an acid–base pair is therefore not trivial to predict, and the experimental determination of how the conjugate bases are bridged by the hydrogen bonds is of high interest.^25−27^

Infrared (IR) action spectroscopy has demonstrated its effectiveness as a technique for experimentally characterizing the structures of ionic clusters, and many such studies have been performed on systems ranging from protonated or deprotonated water clusters to larger biomolecules.^25,28−32^ As a variation, IR action spectroscopy can also be coupled to helium droplets isolation methods to reduce spectral congestion, allowing for a more precise determination of molecular interactions.^33−36^

Among ionic complexation motifs, complexes formally consisting of two anionic bases and a proton [B_1_^–^···H^+^···B_2_^–^]^−^ are especially interesting, as they contain many of the essential interactions, yet being small enough to allow for detailed testing of theoretical descriptions. When B_1_^–^ = B_2_^–^, the potential for the proton can be a double minimum or a single well potential. In the case of B_1_^–^ = B_2_^–^ being HCOO^–^ (formate), the potential has only a single minimum and the proton is equally shared.^33^ For B_1_^–^ = B_2_^–^ = OH^–^ (hydroxide), the potential has two minima, yet the zero point energy is above the barrier, leading to an equally shared proton as well.^37−39^

When the two bases are not the same, the location and dynamics of the proton will depend on the differences in PA and additional factors, leading to either a single or a double minimum potential and an asymmetrical location of the proton. Conventional wisdom would say that, in a first approximation, the proton is located at the site with the highest PA.

One example of such a system is the proton-bound complex of hydrogen sulfate with formate, [HSO_4_^–^···H^+^···HCOO^–^]. Sulfuric acid is widely known for its strong acid character. The difference in p*K*_a_ values of sulfuric and formic acid (−3 and +3.75, respectively), suggests in the case of aqueous solution a strong preference for the proton to be located at the formate moiety. In the case of the gas phase, the PA values are 1449 kJ/mol^40^ for the formate ion and 1295 kJ/mol^41^ for the hydrogen sulfate ion. The difference in PA values of both bases implies that the formate accepting a proton to form formic acid is favored energetically by around 154 kJ/mol.

This simple picture has been challenged in a photoelectron study on [HSO_4_^–^···H^+^···HCOO^–^].^42^ However, in a more recent IR-spectroscopic study, this dimer has been found to have a complex geometry with a proton shared between the hydrogen sulfate and formate moieties, with a preference for location at the formate.^43^

In this work, the proton-bound dimer of the dihydrogen phosphate and formate [H_2_PO_4_^–^···H^+^···HCOO^–^] is interrogated by cryogenic IR action spectroscopy. The difference in p*K*_a_ values of phosphoric and formic acid (+2.15, +3.75, respectively), suggests the proton is located at the formate moiety in the aqueous solution. In the case of the isolated complex in the gas phase, the PA of dihydrogen phosphate (H_2_PO_4_^–^) is 1383 kJ/mol,^44^ 66 kJ/mol lower than the PA of formate, which suggests that also in this complex, the proton should be located in the formate moiety.

## Methods

### Experimental Methods

Gas-phase IR spectra of the phosphoric acid–formate proton-bound dimer at different levels of hydrogen-to-deuterium exchange are measured using helium nanodroplets infrared action spectroscopy. The home-built instrument has been reported in detail in previous publications,^33,34,45^ and only an overview and specific details are provided herein. The ions of interest are generated via nanoelectrospray ionization (nESI), using Pd/Pt-coated pulled borosilicate capillaries fabricated in-house and a solution of 4 mM formic acid (>98%, Sigma-Aldrich Merck, Darmstadt, Germany) and 2 mM phosphoric acid (96%, Sigma-Aldrich Merck, Darmstadt, Germany) in a 1:1 mixture of water and acetonitrile. To substitute exchangeable hydrogen with deuterium atoms, the source region is flooded with D_2_O-saturated nitrogen.

After the transfer of the ions into the vacuum, the dihydrogen phosphate–formate complex ions are isolated by a quadrupole mass filter. The ions are then deflected by 90° by a quadrupole ion bender and injected into a hexapole radio frequency (RF) ion trap. In the trap, ions are confined in radial direction by the effective RF potential and in the longitudinal direction by a weak potential (≈3 V) applied to the entrance and exit lenses of the trap. In this experiment, the housing of the trap is cooled to ≈90 K using a nitrogen gas flow. Before the ions enter the trap, precooled helium buffer gas is introduced to thermalize the ions by collisions.

After pump-out of the buffer gas, the ion trap is traversed by a beam of helium nanodroplets. The nanodroplets are generated by the expansion of helium (≈70 bar) through the cryogenic nozzle (19–23 K) of a pulsed Even-Lavie valve operated at a repetition rate of 10 Hz. The size distribution of helium droplets follows a log–normal distribution and under the here used experimental conditions, this distribution is expected to have a maximum (mode) of 5 × 10^4^ and a mean of 7.1 × 10^4^ He atoms.^46^ The droplets travel at a beam velocity of 500 m/s^47^ and due to their high mass, even relatively small droplets containing only a few hundred helium atoms have a kinetic energy that is higher than the longitudinal trapping potential of the ion trap. When a droplet picks up an ion via mechanical impact, the ion will be cooled to the equilibrium temperature of the droplet (0.4 K), and the ion doped droplet can exit the trap.

After traveling downstream the instrument, the doped droplets are overlapped with the tunable IR light beam of the Fritz Haber Institute infrared free-electron laser (FHI-FEL), which provides IR light in the form of ≈10 μs long macropulses at a 10 Hz repetition rate, consisting of micropulses of ≈5 ps length at a repetition rate of 1 GHz.

When the doped droplets interact with the FHI-FEL light, the resonant absorption of photons can occur. Experimentally, the appearance of bare unsolvated ions is observed at a certain IR wavelength, possibly indicating the complete evaporation of the droplet. Using the parameters of the log–normal distribution above, approximately 10% of the droplets have a size of less than 3.4 × 10^4^ He atoms. Using 5 cm^–1^ as the binding energy for a helium atom to a droplet,^47^ this implies that 1.7 × 10^5^ cm^–1^ of energy, or 110 photons at 1500 cm^–1^ need to be absorbed to completely evaporate those 10% of the doped droplets.

The absorption of the photons can occur sequentially when the energy of each photon is transferred from the absorbing mode, first within the molecule by intramolecular vibrational redistribution (IVR), and then to the helium environment of the droplet. This is followed by the evaporation of helium atoms and the thermalization of the droplet as well as the dopant ion back to the equilibrium temperature of 0.4 K. Such a process can occur many times on the time scale of the FHI-FEL macropulse. Its efficiency depends on the absorption cross-section, the laser fluence, and the relaxation dynamics and rate constants. A more detailed description is given in the Supporting Information.

In the experiment presented here, the bare ions produced after IR irradiation are then deflected by a second quadrupole bender to be finally detected by a time-of-flight mass analyzer. Cold ion infrared spectra are obtained by measuring the mass-to-charge selected signals of the bare ions as a function of the wavenumber.

For the results presented in this work, the IR spectra of the dihydrogen phosphate–formate proton-bound dimer [H_2_PO_4_^–^···H^+^···HCOO^–^] ([FP-H_3_]^−^) and its counterparts with different deuterium isotopic substitutions ([FP-H_2_D]^−^, [FP-HD_2_]^−^, and [FP-D_3_]^−^) are measured in the 800–1650 cm^–1^ range by scanning the photon energy in steps of 1 cm^–1^. The substitution of the exchangeable hydrogens with deuterium in the ion source of the instrument is not complete, and the nondeuterated dimer, two partially deuterated, as well as the fully deuterated complexes, are generated simultaneously. In those experiments, the quadrupole mass filter resolution is intentionally lowered to allow for all four species to be sent to the trap at the same time, which allows for the recording of their IR spectra simultaneously.

Previous studies using the same action spectroscopy technique have shown a nonlinear dependence of the ion signal on the FEL macropulse energy.^48^ Nonetheless, as a first-order correction for variations in IR laser fluence, the intensities in the spectra are divided by the photon fluence. Each spectrum is recorded at least twice and then averaged. Since the intensity depends nonlinearly on the photon fluence, situations can arise where weak transitions are only observed when the signal of strong bands is saturated. Therefore, several sets of spectra are recorded at different FEL macropulse energies. At *high* macropulse energies (>110 mJ), all the vibrational bands are visible, however, stronger bands can be saturated and broadened. Those saturated bands then show no notable intensity differences between them. At *low* macropulse energies of around 30 mJ, most vibrational bands can be observed, and their relative intensities can be compared to predictions from theory. An additional IR beam attenuator is used to record a third set of spectra at *very low* macropulse energies (∼15 mJ). At such experimental conditions, only strong intensity bands are visible (see Supporting Information). In all macropulse energy regimes, the IR spectra of the FP nondeuterated dimer, as well as the three different deuterated species, are recorded simultaneously. To better compare the recorded intensities of those different species, the intensities of their spectra are scaled to their relative ion currents injected in the trap, and the maximum in the four spectra at the given experimental conditions is set to 100.

### Theoretical Methods

A search through the conformational space is made using CREST^49^ and the GFN2-xTB method,^50^ resulting in three different conformers. Those are further optimized, and their harmonic IR frequencies are calculated using the Gaussian 16 software package^51^ at different levels of theory. DFT calculations are performed using the B3LYP functional with the GD3BJ^52^ dispersion correction and the Jul-cc-pV(T + d) basis set.^53^ The selection of the Jul-cc-pV(T + d) basis set is made, as Barone et al.^54^ reported that the use of partially augmented basis sets including an additional set of d functions may improve the computations on third-row p-block elements. Additionally, IR frequencies are calculated within the GVPT2 (generalized second-order vibrational perturbation theory)^55^ anharmonic approximation. For comparison to the experiment, harmonic frequencies are scaled by a factor of 0.975, which is the computed average of the anharmonic to harmonic frequency ratio (see Supporting Information). All theoretical spectra are convoluted with Gaussian functions with a full width at half-maximum (fwhm) of 0.4% of the wavenumber, which is the value of the bandwidth of the FHI-FEL in most experiments. Additional computations using different methods and basis sets are performed for comparison, and those results can be found in the Supporting Information.

## Results and Discussion

### Experimental IR Spectra of the Proton-Bound Dimer of Dihydrogen Phosphate and Formate

**Figure 1:**
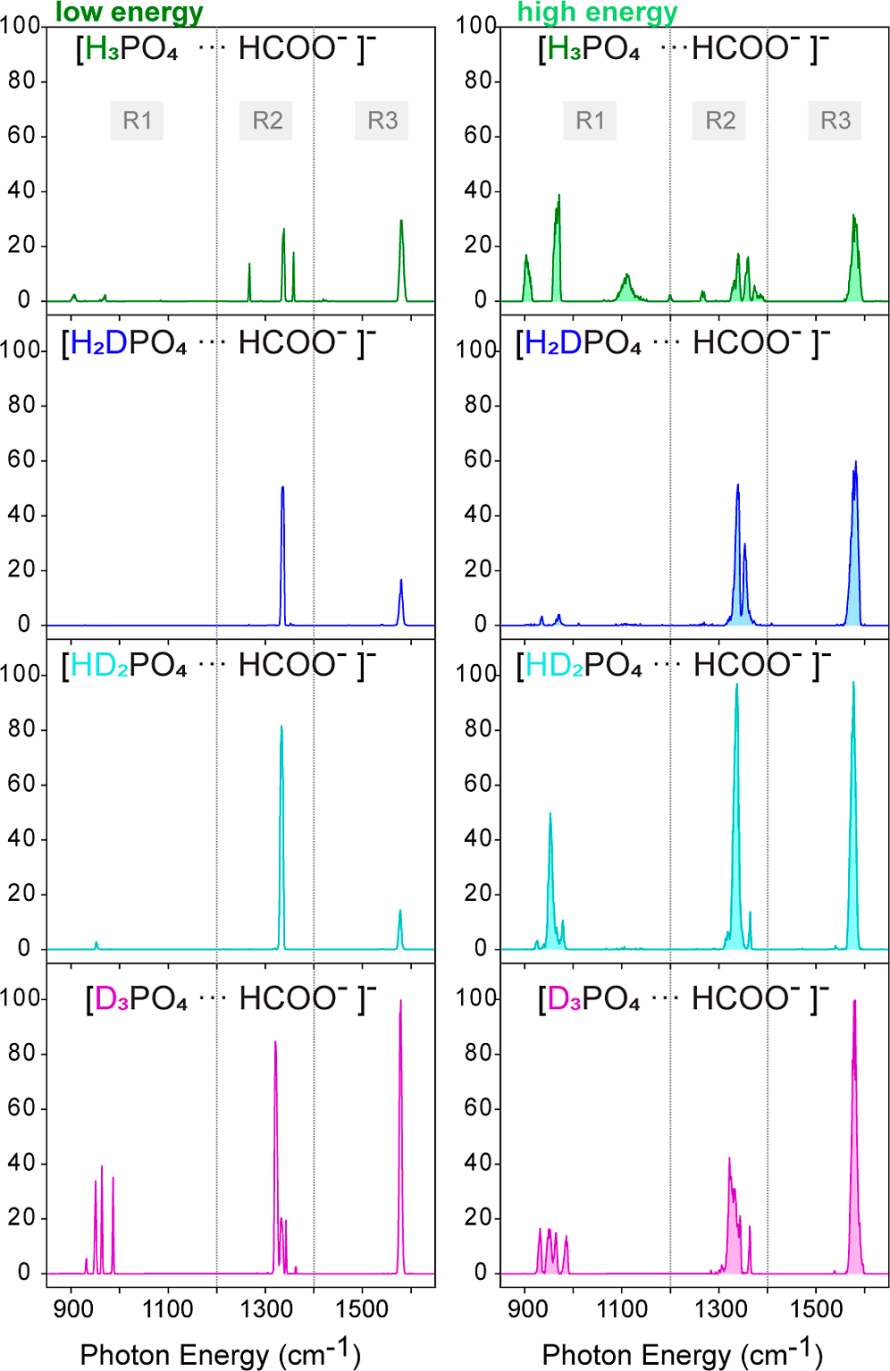
Experimental IR action spectra of the dihydrogen phosphate–formate proton-bound dimer [FP-H_3_]^−^ and its isotopically substituted counterparts [FP-H_2_D]^−^, [FP-HD_2_]^−^, and [FP-D_3_]^−^. The spectra measured at *high* FEL macropulse energy are filled in light colors. Three main spectral regions (R1, R2, and R3) are defined in the spectra of all species.

The cryogenic IR spectra of the dihydrogen phosphate–formate proton-bound dimer ([FP-H_3_]^−^) and its isotopically substituted counterparts ([FP-H_2_D]^−^, [FP-HD_2_]^−^, [FP-D_3_]^−^), are recorded simultaneously using *low* and *high* FEL macropulse energies, and are shown in Figure 1. The intensity values on the *y*-axis represent the percentage of the maximum intensity recorded for each set of spectra. In all experiments, the [FP-D_3_]^−^ exhibits the most intense transitions. As discussed in the Experimental Methods section, at *high* FEL fluences, saturation in spectra can occur. However, when measuring at *low* FEL macropulse energy regime, the relative intensities of the spectra can be compared. Interestingly, the intensities of the nondeuterated dimer ([FP-H_3_]^−^) transitions are about 3 times less intense compared to the fully deuterated ([FP-D_3_]^−^) ones.

Three main spectral regions (R1, R2, and R3) can be identified in the spectra of the four species (Figure 1). In the region above 1400 cm^–1^ (R3), at all levels of deuteration, a single band is observed at ≈1580 cm^–1^. For neutral formic acid in the gas phase, a strong C=O stretching IR absorption band is observed at 1767 cm^–1^ for cis and at 1808 cm^–1^ for trans formic acid.^56^ For isolated formate anions, a strong band that stems from the COO^–^ antisymmetric stretching vibration is observed near 1623 cm^–1^. The band at ≈1580 cm^–1^ is an indication that the proton is not located in the formate moiety. In the region between 1200 and 1400 cm^–1^ (R2), a group of bands is observed for all four species. Previous IR experiments of microhydrated dihydrogen phosphate clusters in the gas phase report the presence of bands assigned to P=O stretching vibrations between 1000 and 1350 cm^–1^.^21^

The spectral signatures of the four species in the region below 1200 cm^–1^ (R1) vary significantly. In the *low* FEL energy spectrum of the nondeuterated dimer, two weak features are visible below 1000 cm^–1^ (905 and 968 cm^–1^). Those bands are no longer visible in this region after one H/D exchange. When two hydrogen atoms are replaced by deuterium a very weak band appears at 955 cm^–1^. In contrast, the spectrum of the fully deuterated dimer shows 4 sharp and intense bands between 900 and 1000 cm^–1^. For phosphoric acid ions in the gas and in the condensed phase, experiment and theory show the presence of ν(P–O) and the PO–H out-of-plane bending modes between 600 and 1200 cm^–1^.^21,57^

As previously discussed in the Methods section, when using *high* FEL macropulse energies, additional bands are observed, however, some of the intense bands can become broader and their intensities saturated. In contrast, at *low* FEL macropulse energies, only transitions with relatively high absorption cross sections are observed and the corresponding spectra contain fewer and narrower bands. A further difference between the *low* and *high* macropulse energy spectra is that for [FP-H_3_]^−^ at *high* macropulse energy, an unusual broad band is observed near 1110 cm^–1^. This band is not visible in the low energy spectrum and does not have a counterpart in position or width in the spectra of the deuterated species. This transition will be discussed later in the manuscript. In the spectrum of the nondeuterated dimer [FP-H_3_]^−^ recorded using *high* macropulse energies, the low-frequency region (R1) shows again the presence of two bands at 905 and 968 cm^–1^. For [FP-H_2_D]^−^ two very weak bands in the same region are visible. The observation is similar for [FP-HD_2_]^−^, where weaker transitions are observed in the R1 region as well. In the case of the fully deuterated species [FP-D_3_]^−^, the R1 region shows a similar set of bands as in the *low* energy spectrum, but their widths become, however, larger. In the context of the strong differences observed in the spectra of each species, it is important to remark again that the IR spectra of [FP-H_3_]^−^ and all its isotopically substituted counterparts ([FP-H_2_D]^−^, [FP-HD_2_]^−^, [FP-D_3_]^−^) in both energy regimes are recorded simultaneously.

### Quantum Chemistry Calculations

**Figure 2:**
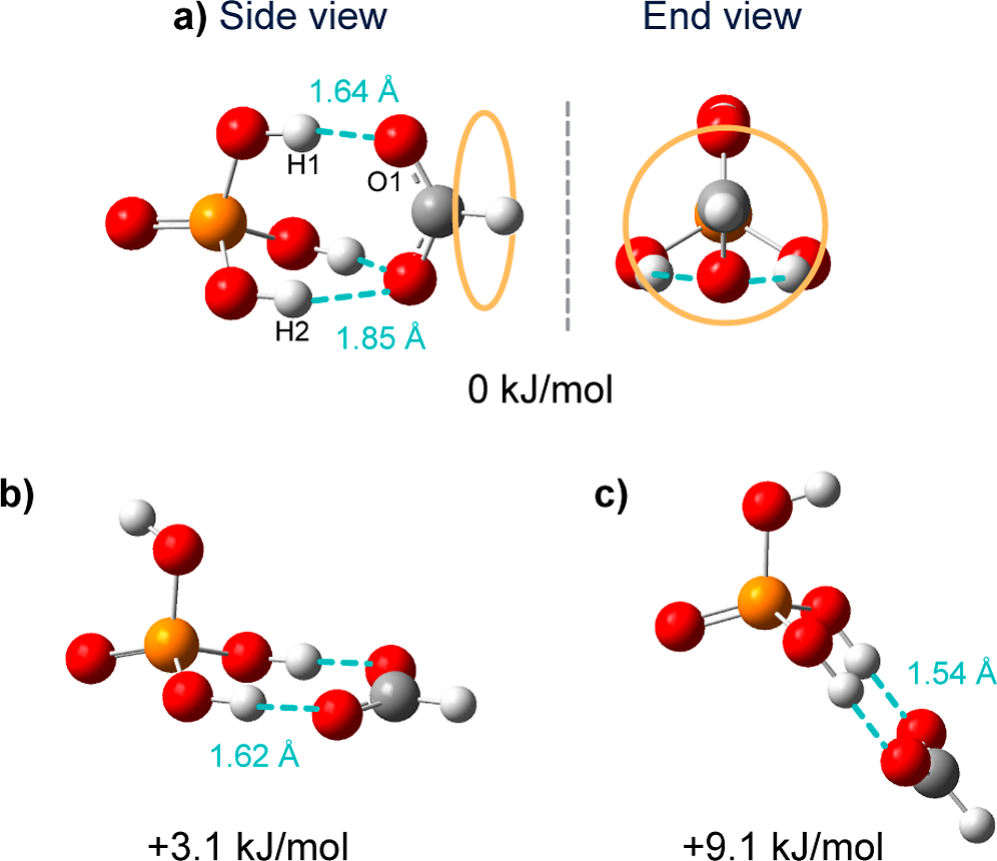
Optimized structures and relative electronic energies (EE + ZPE) of the proton-bound dimer phosphoric acid–formate ([H_2_PO_4_^–^···H^+^···HCOO^–^]) computed at the B3LYP-D3(BJ)/Jul-cc-pV(T + d)Z level of theory.

When performing a search through the structural landscape, three local minima for the [FP-H_3_]^−^ dimer are found (Figure 2). All three structures have *C*_*s*_ symmetry where all the acidic protons are localized in the phosphate moiety. Interestingly, no minima were found corresponding to a structure in which the proton is situated on the formic acid moiety. In the lowest energy structure (a), all the phosphoric acid hydrogen atoms interact with the oxygen atoms on the formate unit. One strong hydrogen bond with a bond length of 1.64 Å (H1–O1) and two weaker and equivalent 1.85 Å long hydrogen bonds between H2/H3 and the second oxygen of formate are present. Structures (b) and (c) exhibit only two hydrogen bonds, which resemble the hydrogen bonding motif in the most stable conformer reported for the proton-bound dimer of hydrogen sulfate and formate.^43^ The main difference between the structures (b) and (c) is in the third hydrogen atom on the phosphoric acid unit, which is not involved in a hydrogen bond. In the case of structure (b), this hydrogen atom is pointing backward with respect to the formate, while it is pointing forward in structure (c).

The relative energies of the three structures are calculated at different levels of theory, yielding similar results (see Supporting Information). At all levels of theory, structure (a) is lowest in energy. The energy of structure (b) is +3.1 kJ/mol higher, followed by (c) [+9.1 kJ/mol relative to (a)], making those two structures unlikely to be present in the gas phase under our experimental conditions. Further, the comparison between the experimental spectrum and the theoretical spectra for structures (b) and (c) shows a poor match (Supporting Information) and further on we therefore only consider structure (a).

**Figure 3:**
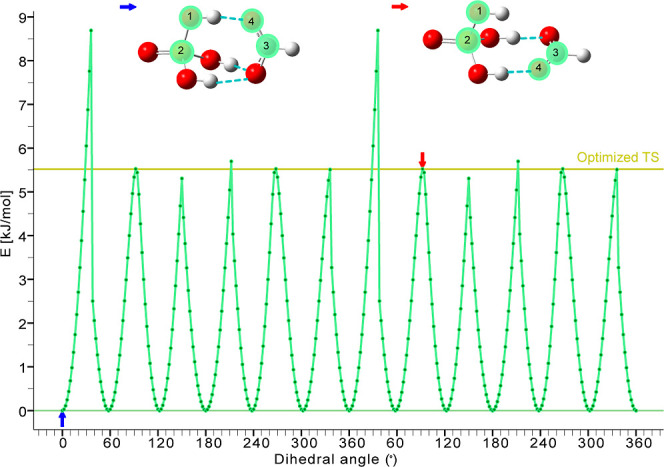
Relaxed potential energy scan [B3LYP-D3(BJ)/Jul-cc-pV(T + d)Z] of the rotation of the formate unit with respect to the phosphoric acid with the dihedral angle formed by O–P–C–O1 as the scan variable. The minimum energy structures (blue arrow) occurring every 60° correspond to structure (a). The maxima correspond to transition state structures with only two hydrogen bonds which are 5.6 kJ/mol above the ground state.

For hydrogen-bonded complexes, internal dynamics might also contribute to the vibrational features, even at low temperatures. Structure (a) has six symmetry equivalent structures that differ only by the rotation of the formate unit, approximately around the C–H axis. Those structures are separated by transition states that might be low in energy. To explore this degree of freedom, we performed a relaxed potential energy scan at the B3LYP-D3(BJ)/Jul-cc-pV(T + d)Z level of theory, and the results are shown in Figure 3. The scan variable is the dihedral angle between atoms 1, 2, 3, 4 (see Figure 3) and the scan is performed for two revolutions. As expected, the scan shows the presence of six equivalent minima. The first barrier is higher, which is due to an artifact resulting from the definition of the dihedral angle, the remaining barrier heights are around 5.6 kJ/mol. The structure at the barrier is used as a starting point for a transition state optimization at the B3LYP-D3(BJ)/Jul-cc-pV(T + d)Z level of theory. The resulting transition state structure is found to have one and only one imaginary normal mode, is of *C*_*s*_ symmetry, and is shown with the red arrow in Figure 3. The structure is essentially unchanged and the relative energy is again 5.6 kJ/mol above the minimum. This energy is likely much higher than the zero point energy in this coordinate and further, as this rotation coordinate involves the motion of heavy atoms, tunneling is highly unlikely. We therefore conclude that internal dynamics in this coordinate will not play a role in our spectra.

### Comparison of the Experimental and Theoretical Spectra

**Figure 4:**
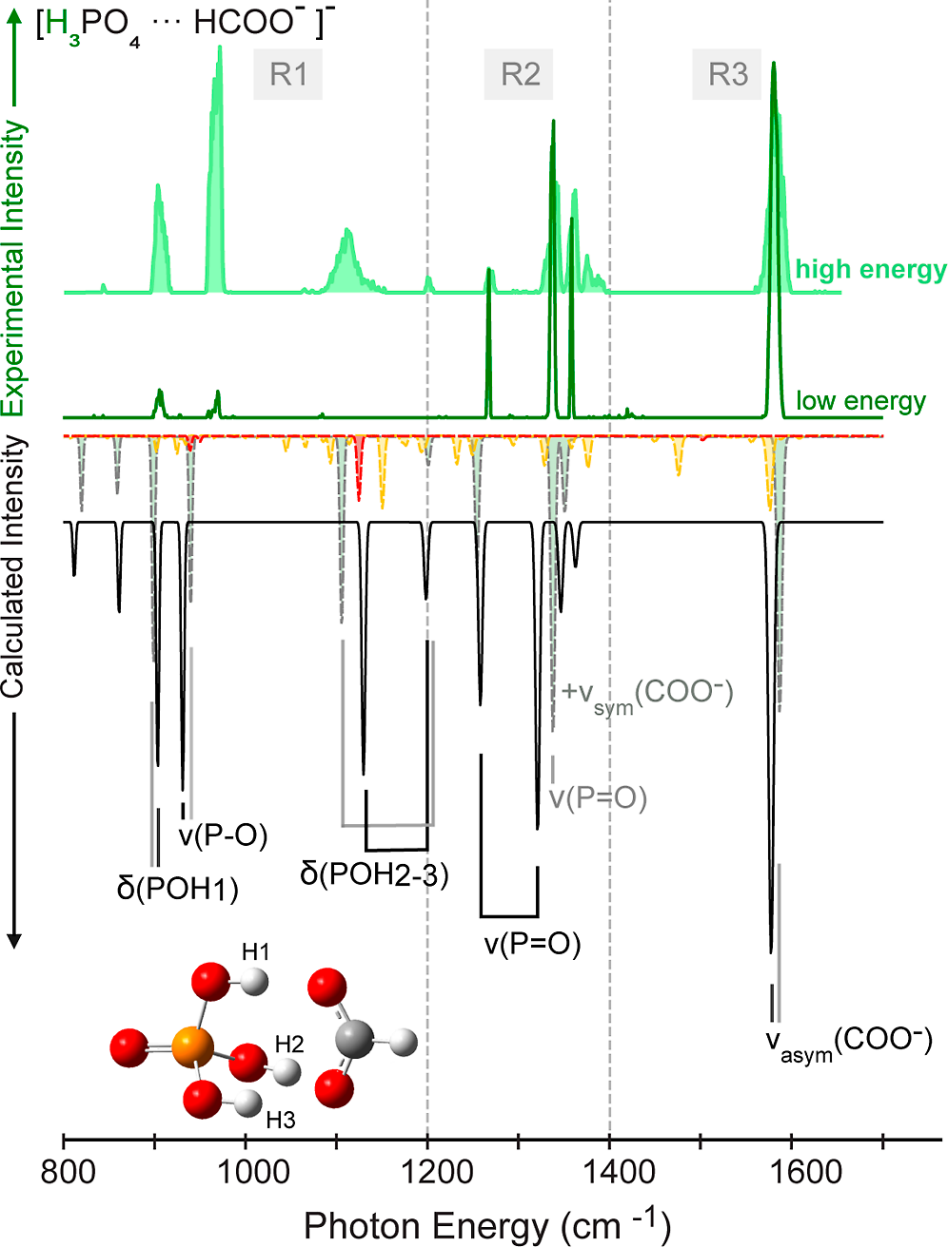
Experimental IR action spectrum of the [FP-H_3_]^−^ complex at *high* (filled light green) and *low* (dark green) FEL macropulse energy in comparison with the calculated IR spectra of structure (a) (Figure 2) computed at the B3LYP-D3(BJ)/Jul-cc-pV(T + d)Z level of theory. The calculated spectrum in the harmonic approximation (scaled by 0.975) is shown in black. The calculated spectrum in the anharmonic approximation is shown in dashed lines. The spectrum comprises bands stemming from fundamental modes (gray), combination bands (yellow), and overtones (red).

Figure 4 shows the experimental spectra of the [FP-H_3_]^−^ at *low* and *high* FEL macropulse energy together with spectra calculated at the harmonic approximation as well as with anharmonic corrections at the GVPT2 level, both calculated at B3LYP-D3(BJ)/Jul-cc-pV(T + d)Z level of theory. The harmonic frequencies are scaled by 0.975. This factor is derived by taking the average of the ratio between the calculated anharmonic and harmonic frequencies of all isotopologues considered in the 800–1700 cm^–1^ range. This scaling factor is close to a literature value of 0.962.^58^ Scaling all harmonic frequencies by 0.975 brings them uniformly close to the anharmonic values, indicating that anharmonic effects are not too large, so GVPT2 is a valid method for this complex, and the harmonic approximation provides a good zero-order description. This is in sharp contrast to other hydrogen-bound acid–base complexes where the harmonic approximation is very poor for certain vibrations and perturbation treatment does not yield satisfactory results.^59^

The normal modes at the harmonic approximations have been visualized in order to correlate them to the motions of (groups of) atoms. Most modes are found to be delocalized and highly coupled, making such an assignment difficult, and the labeling of these modes is made according to their main vibrational contribution. In the R3 region, the calculated transition at a (scaled harmonic) frequency of 1585 cm^–1^ can be easily assigned to result from mainly ν_antisym_(COO^–^) stretching vibration. In the R2 region, the assignment of the vibrational band gets more difficult. The transitions calculated at 1326 and 1262 cm^–1^ both contain a large fraction of P=O stretching motion, with the 1326 cm^–1^ transition being mixed with ν_sym_(COO^–^) stretching motion and the 1262 cm^–1^ transition also involving δ(PO–H1) motion. In the R1 region, two transitions located at 1202 and 1132 cm^–1^, correspond to relatively localized bending modes of the two less strongly hydrogen-bonded hydrogen atoms H2 and H3 δ(PO–H2,H3). Between 900 and 1000 cm^–1^, two main transitions at 932 and 904 cm^–1^ result from the motion of the O–H1 moiety, namely ν(P–O) and δ(PO–H1) vibrations, respectively.

When comparing the spectrum recorded at *low* FEL energy with theory one can note that above 1200 cm^–1^ (R2 and R3), peak positions and their relative intensities match very well. Below 1200 cm^–1^, almost all calculated bands have counterparts in the experimental spectra. The relative intensities observed at *low* FEL energy are, however, lower than expected from theory. We will address this point further below. Nonetheless, the calculated scaled harmonic and the anharmonic spectra for structure (a) predict the positions and relative intensities of the vibrational bands overall quite well (Figure 4). In comparison, the match to calculated spectra of structures (b) and (c) is poor (Supporting Information), giving strong support to structure (a) as the one being present in the experiment.

**Figure 5:**
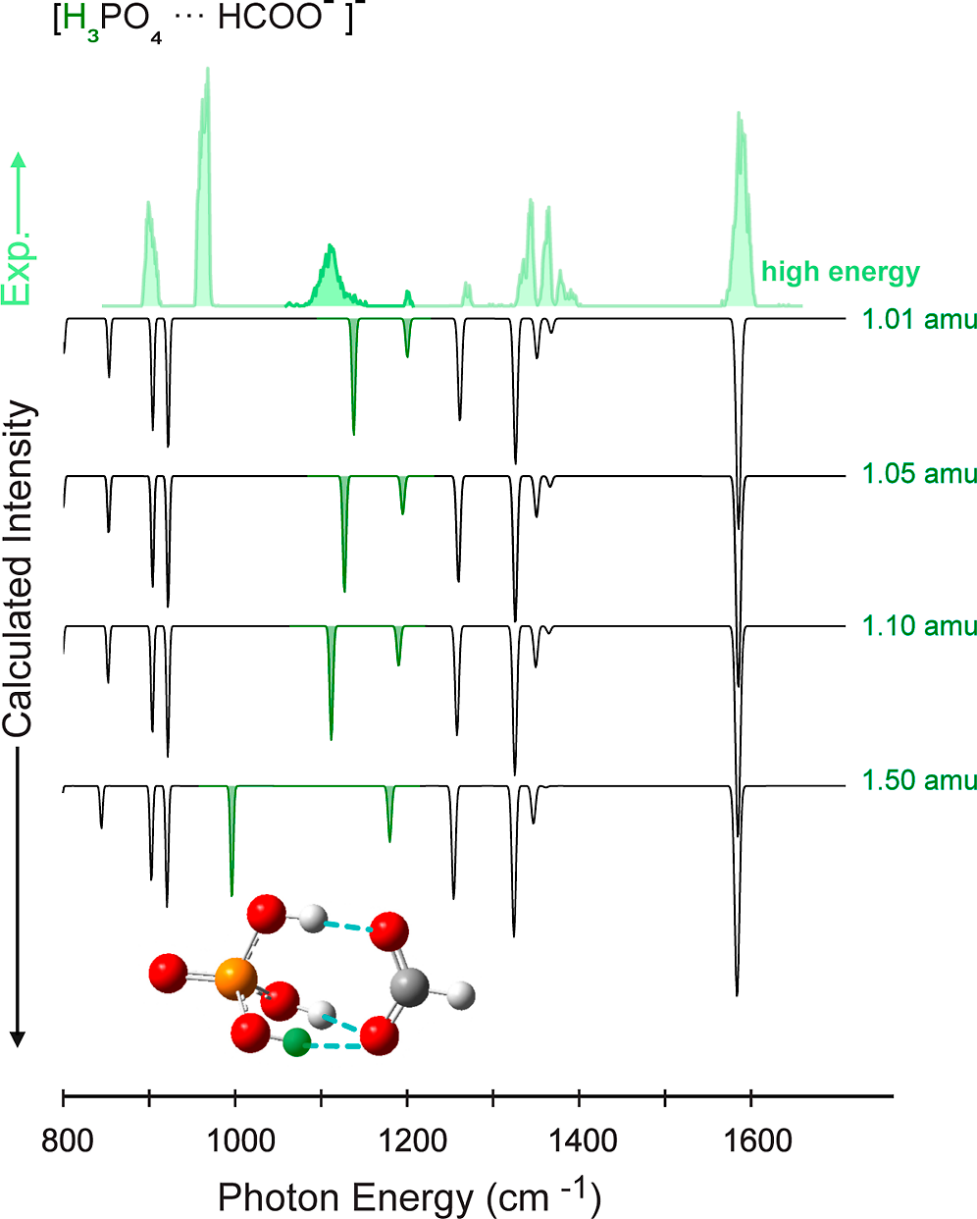
Experimental infrared spectra of the [FP-H_3_]^−^ measured using *high* macropulse energies (light green) compared to the harmonic theoretical spectra (B3LYP-D3(BJ)/Jul-cc-pV(T + d)Z, scaled by a factor of 0.975) (black line), modifying the mass of one of the weakly bound hydrogens (green highlight) by 1, 5, 10, and 50%.

A peculiar band is the weak and broad feature observed in the experiment at 1110 cm^–1^, only visible at *high* FEL macropulse energy (Figure 4). Theory predicts near that position at 1132 cm^–1^ a quite strong transition. As stated above, it can be assigned to highly localized δ(PO–H2,H3) bending motion. When looking at the anharmonic spectrum (dashed lines), we can see that there is one fundamental band predicted between 1100 and 1200 cm^–1^, which is close in frequency to an overtone (red) and to a combination band (yellow). These three transitions involve the motion of the H2 and H3 hydrogen atoms. Energy redistribution between these three modes could occur. Also, the localized bending motions of H2 and H3 might be vulnerable to perturbations and inhomogeneous broadening resulting from the interaction with the helium surrounding. The sensitivity of such a vibration to variations in the surroundings could be qualitatively assessed by artificially increasing the mass of the hydrogen atoms involved. The result of such a calculation is shown in Figure 5, where the spectrum is calculated with the mass of one of the hydrogen atoms (marked in green in the structure of Figure 5) increased by 1, 5, 10, and 50%. Similar calculations separately changing the masses of H1 and both H2 and H3 are shown in the Supporting Information (S10, S11). In Figure 5, one can observe that in the resulting spectra, most bands do not change. The exceptions are the two bands marked in green. The band at 1110 cm^–1^ shifts strongly, even with comparatively small changes in the mass of the corresponding hydrogen atom. On the other hand, the change on the band near 1200 cm^–1^ is less pronounced. A slowly fluctuating helium environment that interacts with the atoms in the complex could have a similar effect as randomly varying the masses by a small amount, and a mode that is highly sensitive to the atomic masses might therefore appear broadened. Such a simple picture might explain why the band near 1110 cm^–1^ appears broad while the band near 1200 cm^–1^ remains narrow.

For the partially deuterated complexes [FP-H_2_D]^−^ and [FP-HD_2_]^−^, two possible isotopomers (isomers that differ by the location of the isotopic substitution) exist, which differ in energy by the difference in their zero point energy. This difference is dominated by the differences in zero point energy in the O–H and O–D stretch frequencies. The O–H2 and O–H3 stretch vibrational frequencies (see Figure 2) are about 400 cm^–1^ higher, compared to the O–H1 stretch vibrational frequency. Upon deuterium substitution, therefore, the energy of the complex is lowered most when the H2 and H3 hydrogen atoms are replaced by deuterium. Therefore, for [FP-H_2_D]^−^, the lowest energy isotopomer will have the deuterium atom in either H2 or H3 position, and the higher energy isotopomer will have the deuterium atom in H1 position. For [FP-HD_2_]^−^, the lowest energy isotopomer will have the hydrogen atom in H1 position and the higher energy isotopomers will have the hydrogen atom in H2 or H3 position. In both cases, the energy difference between the high and low energy isotopomer is small and calculated to be ≈0.25 kJ/mol.

**Figure 6:**
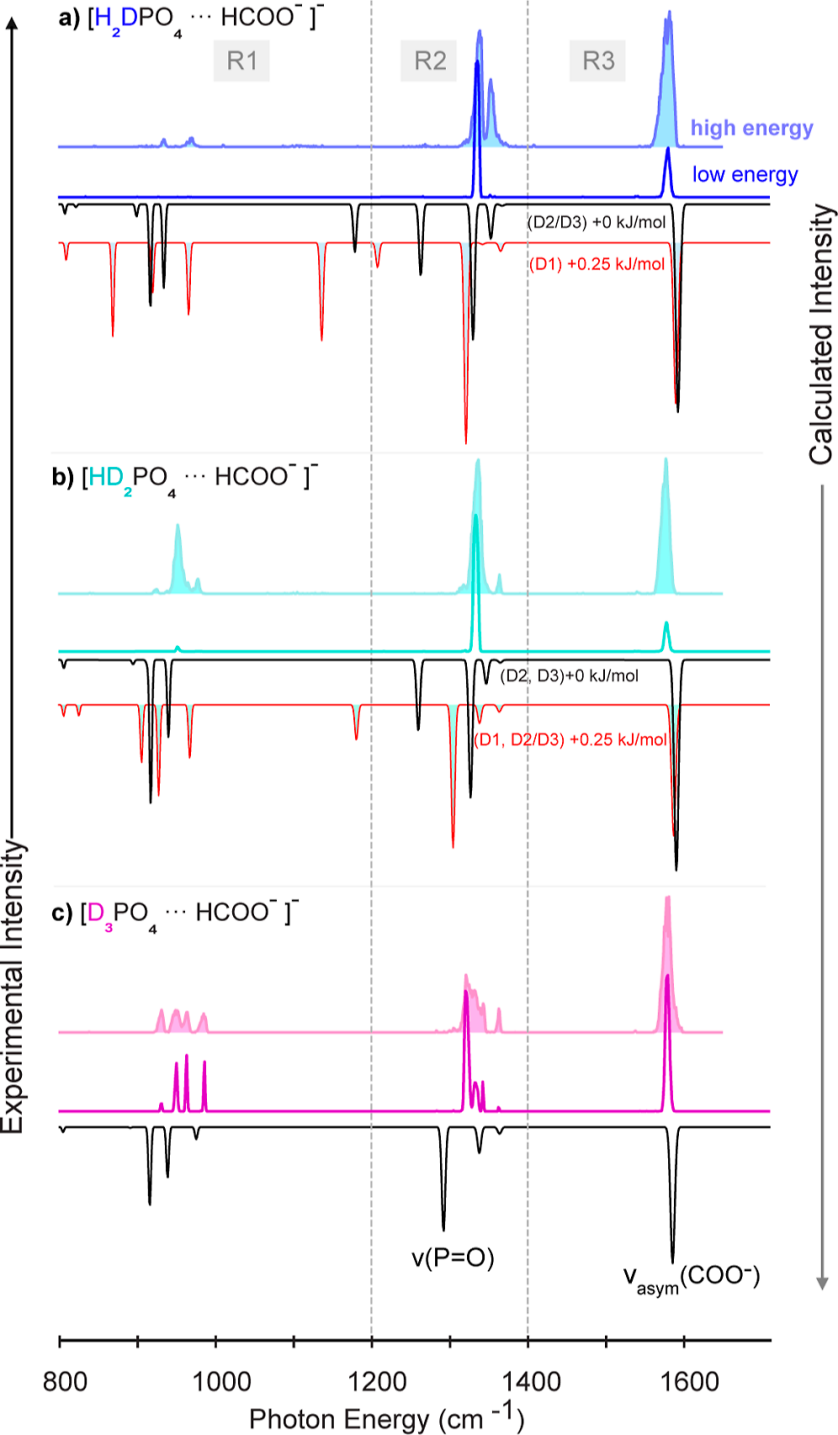
Experimental IR action spectra of [FP-H_3_]^−^ complexes with varying degrees of hydrogen by deuterium substitution, (a) [FP-H_2_D]^−^, (b) [FP-HD_2_]^−^, (c) [FP-D_3_]^−^, measured at *high* (filled spectra) and *low* FEL macropulse energy. In comparison, predicted harmonic spectra (0.975 scaled, black) of the lowest minimum structure computed at the B3LYP-D3(BJ)/Jul-cc-pV(T + d)Z level of theory are shown. In the case of the partially deuterated species the computed spectra of the high-energy isotopomers are shown in red.

In Figure 6, the experimental *high* and *low* FEL energy spectra of the deuterated species ([FP-H_2_D]^−^, [FP-HD_2_]^−^, and [FP-D_3_]^−^) are shown and compared to the harmonic theoretical spectra. The comparison with the spectra calculated in the anharmonic approximation is shown in the Supporting Information. For the partially deuterated complexes, the calculated spectra for both isotopomers are shown. In all experimental and calculated spectra, intense antisymmetric ν(COO^–^) stretching modes are observed in R3. Their band positions undergo only small shifts upon deuteration and show little variation for the different isotopomers.

Further, in all the spectra an intense band stemming from ν(P=O) stretching vibrations is predicted between 1300 and 1400 cm^–1^. This band is accompanied by weaker sidebands, which are assigned from visualization to contain symmetric ν(COO^–^) stretching vibrations. For the partially deuterated species, experiments show intense bands that are close in position to those predicted for the lowest energy isotopomer. In the case of the fully deuterated complex, the agreement is less good and the theoretical transition is more shifted to the red.

For the partially deuterated species, theory predicts bands between 1100 and 1250 cm^–1^. Their positions are very different for the two isotopomers. Experimentally, no bands are observed in that range, even at *high* FEL macropulse energies. Below 1000 cm^–1^ multiple bands are predicted. Experimentally, for [FP-H_2_D]^−^ and [FP-HD_2_]^−^, only very weak transitions are observed, and in the case of the [FP-H_2_D]^−^, only when using *high* energies. In contrast, for [FP-D_3_]^−^ in the same region, moderately strong transitions are observed, however, the band positions only match qualitatively.

An interesting question is why in the case of the partially deuterated species, fewer bands are present in the experimental spectra than in those predicted by theory. In both, the [FP-H_2_D]^−^ and the [FP-HD_2_]^−^ cases, the energy difference between the two isotopomers is very small. As discussed in the Methods section, when the complex is captured by a helium droplet in the ion trap, rapid cooling (shock freezing) of the complex to 0.4 K is expected. The Boltzmann distribution of isotopomers at the trap temperature of 90 K should be retained and yield almost equal populations of both isotopomers. Contrary to this expectation, the experimental spectra do not appear to be the superposition of the spectra of the two isotopomers. One possible explanation is related to the multiple photon absorption characteristics of our experiment, which requires the absorption of >100 photons in order to yield an ion signal. After the resonant absorption of a photon, the internal energy of the complex is increased. The isomerization between the isotopomers likely involves the rotation of the formate unit with a calculated barrier of 468 cm^–1^ (see Figure 3). When the photon energy is above this barrier, this isomerization can occur. As the energy difference between the isotopomers is small, such an isomerization can occur in both directions. In the case that this newly formed isotopomer does not absorb at this particular photon energy, the excitation laser is no longer resonant and the absorption process stops, impacting the observed ion signal.

If we compare the calculated spectra of the different isotopomers, we can notice that for the ν(COO^–^) stretching modes in the R3 region (at around 1580 cm^–1^) and the ν(P=O) modes near 1330 cm^–1^, their positions are relatively close for both isotopomers. Below 1250 cm^–1^ on the other hand, the positions of the bands differ considerably. In case excitation occurs near 1580 cm^–1^, even if isomerization during the sequential multiple photon absorption process occurs, the absorption of photons can continue. Near 1320 cm^–1^, bands do shift, however, weaker bands at the side of the main bands could allow for continued absorption and repopulation. When exciting below 1250 cm^–1^, very little or no spectral overlap is given and isomerization leads to reduced photon absorption.

**Figure 7:**
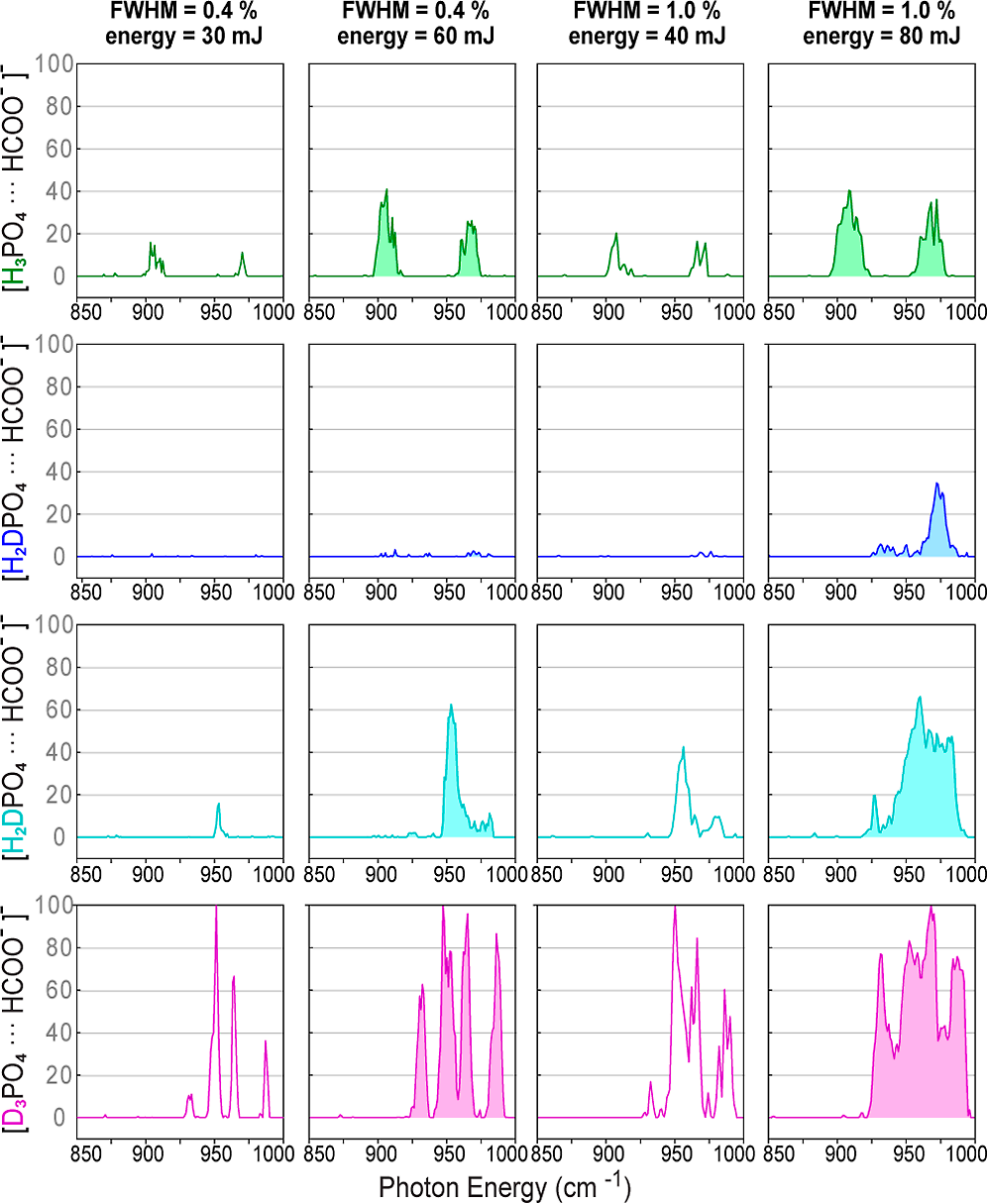
Low-frequency range of the experimental IR action spectra of [FP-H_3_]^−^, [FP-H_2_D]^−^, [FP-HD_2_]^−^, and [FP-D_3_]^−^ measured simultaneously using different FEL bandwidths and macropulse energies.

**Figure 8:**
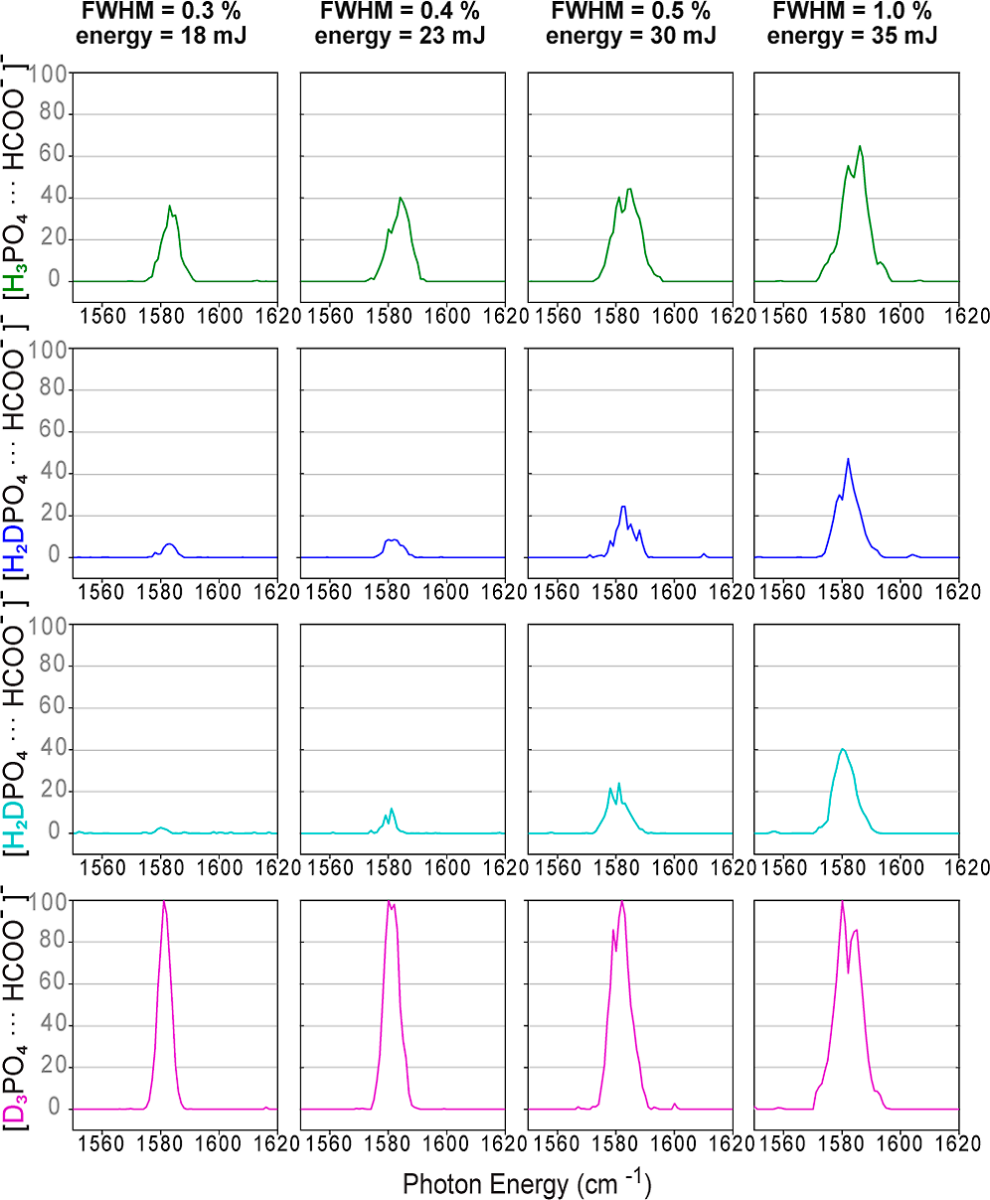
High-frequency range of the experimental IR action spectra of [FP-H_3_]^−^, [FP-H_2_D]^−^, [FP-HD_2_]^−^, and [FP-D_3_]^−^ measured simultaneously using different FEL bandwidths and macropulse energies.

To better understand the excitation process, the dependence of the experimental spectra on the FEL bandwidth and macropulse energy is investigated (Figures 7 and 8). Again, the four species are isolated together in the trap and their spectra are acquired simultaneously. In the low-frequency range (Figure 7) it can be seen that in the cases of the fully hydrogenated ([FP-H_3_]^−^) and the fully deuterated dimer ([FP-D_3_]^−^), the same bands are observed in the spectra. The relative intensities and bandwidths are, however, dependent on the FEL parameters. In the spectra of [FP-D_3_]^−^, a high macropulse energy (filled spectra) results in saturation, causing the four bands to appear at almost of the same intensity. In the case of the partially deuterated species ([FP-H_2_D]^−^ and [FP-HD_2_]^−^), the spectra in this low-frequency region are strongly dependent on the FEL parameters (Figure 7). For [FP-H_2_D]^−^, vibrational transitions in this region are only observable with large FEL bandwidth (fwhm = 1.0%) and high macropulse energy. In the case of [FP-HD_2_]^−^ at narrow bandwidth (fwhm = 0.4%) and low FEL macropulse energy, a weak transition can be observed at 950 cm^–1^. This band gains in intensity and is accompanied by additional bands when increasing the FEL bandwidth and energy. These observations suggest that the appearance of the spectra of the partially deuterated species ([FP-H_2_D]^−^ and [FP-HD_2_]^−^) is indeed strongly influenced by the coincidental spectral overlap between the two different isomers. Spectral broadening causes additional overlap, thereby increasing the number of photons that can be absorbed.

In Figure 8 the high-frequency portion of the spectra of [FP-H_3_]^−^, [FP-H_2_D]^−^, [FP-HD_2_]^−^, and [FP-D_3_]^−^ as a function of bandwidths is shown. In this region, only one vibrational band [ν(COO^–^)] is observed, and the calculations predict for this band approximately the same intensity values at all levels of deuteration. For those spectra, the macropulse energies are set to low values to avoid saturation. In the cases of the partially deuterated species ([FP-H_2_D]^−^ and [FP-HD_2_]^−^), the intensity of this band almost vanishes when using narrow bandwidth (fwhm = 0.3%), and grows as the bandwidth gets larger. This is an indication that although the transition frequencies of the two isotopomers are similar, an increase in bandwidth still causes an enhanced absorption. Further, as this transition is at higher photon energies and therefore higher above the barrier, isomerization might play a more important role.

It can also be observed that the intensities of the transition of the nondeuterated FP dimer are significantly lower, compared to its fully deuterated counterpart. This is not predicted by theory, where the intensities of the vibrational transitions of [FP-H_3_]^−^ and [FP-D_3_]^−^ do not differ significantly. Further, unlike in the case of the partially deuterated species, interconversion dynamics will not occur. Then, what could be the cause of this systematic difference in intensities? One possible explanation could be given by the relaxation dynamics. An efficient transfer of photon energy to evaporated helium atoms requires fast IVR and transfer of energy to the helium environment (see Supporting Information). When the associated time constant for such a process is on the order (or slower than) the interaction time with the light, this relaxation might be rate limiting for the amount of energy transferred to helium evaporation. As the system considered here is small, relaxation could be comparatively slow and play a role. A difference between [FP-H_3_]^−^ and [FP-D_3_]^−^ is the density of vibrational states (DOS) of the two systems, which will be higher for [FP-D_3_]^−^. This could lead to faster relaxation and a more efficient coupling to the helium surrounding, which is translated into more signal observed. This aspect, as well as the possible interconversion dynamics, will be further investigated in the future by performing 2-color IR excitation experiments.

## Conclusions

Here, we studied the proton-bound complex of dihydrogen phosphate and formate at various levels of hydrogen to deuterium exchange using cryogenic IR action spectroscopy. The spectra clearly show the presence of an antisymmetric ν(COO^–^) stretching mode, indicating that all three exchangeable protons are with the phosphate unit. This is in contrast to expectations, which would suggest that the stronger phosphoric acid would transfer a proton to the formate unit. However, quantum chemical calculations support the interpretation that the structure observed in the experiment has all three exchangeable protons at the phosphate. This structure is favored because the charge is localized on the oxygen atoms of the formate anion, which interact with the hydrogen atoms of the phosphoric acid. The interaction with the formate unit occurs then via one strong and two equal and weaker hydrogen bonds. A transfer of a proton to the formate unit would give a formal negative charge to the phosphate. This charge would be delocalized between the two oxygen atoms that are not formally attached to a proton, making the structure unfavorable. The experimental IR spectra are compared with harmonic and GVPT2 anharmonic calculations for this structure. In both cases, the agreement between experiment and theory is good, indicating that the system only behaves weakly anharmonic and that no large amplitude hydrogen atom dynamics take place.

In the experimental IR spectra, the transitions of [FP-H_3_]^−^ are observed to be approximately three times weaker than those of [FP-D_3_]^−^. This observation could be an effect of the vibrational relaxation dynamics, due to an increased density of vibrational states for [FP-D_3_]^−^.

In the case of the partially deuterated species ([FP-H_2_D]^−^ and [FP-HD_2_]^−^), two isomers with H and D at different positions are possible. Their energy difference is given by the difference in zero point energy and is very small. Experimentally, the sum of the spectra of the two isotopomers is not observed. A comparison with theory as well as experiments using different FEL bandwidths (fwhm = 0.3–1.0%) and FEL macropulse energy regimes suggest that an interconversion process between these two isotopomers could take place inside the He droplet after photon excitation. Then, only bands having a significant spectral overlap with those of the other isotopomers would be observable in our experiment.

One of the differences when comparing the experimental and theoretical spectra of the [FP-H_3_]^−^ is the absence of a particular strong band, predicted to be at ≈1110 cm^–1^, corresponding to the highly localized δ(PO–H,H) bending motion. When measuring the spectra using *high* macropulse energies, a weak and surprisingly broad transition at ≈1110 cm^–1^ is observed and it is suggested that the band is inhomogeneously broadened due to a helium environment that changes on the time scale of the FEL macropulse.

## Supporting Information Available

Excitation of a Two-level System in Helium Droplets; additional Experimental Spectra; scaling Factor; relative Energies at different levels of theory; comparison of the experimental vs the harmonic calculated spectra at different levels of theory; effective mass of acidic hydrogens; comparison of the experimental spectra of the deuterated species to the spectra calculated in the anharmonic approximation (PDF)
