# Supplementary material for: Investigation of the Proton-Bound Dimer of Dihydrogen Phosphate and Formate Using Infrared Spectroscopy in Helium Droplets
Source: J Phys Chem A. 2024 May 21;128(22):4456–66. doi: 10.1021/acs.jpca.4c01632 (PMC11163467; doi:10.1021/acs.jpca.4c01632)
Supplement: Supplementary file 1 — jp4c01632_si_001.pdf [file jp4c01632_si_001.pdf]

Investigation of the Proton-Bound Dimer of Dihydrogen Phosphate and Formate using Infrared Spectroscopy in Helium Droplets

Supplementary Information

América Y. Torres-Boy,[†] Martín I. Taccone,[†] Carla Kirschbaum,[†,‡] Katja Ober,[†] Tamar Stein,[¶] Gerard Meijer,[†] and Gert von Helden[*,†]

[†]Fritz Haber Institute of the Max Planck Society, 14195 Berlin, Germany
[‡]Institute of Chemistry and Biochemistry, Freie Universität Berlin, 14195 Berlin, Germany
[¶]Institute of Chemistry and Fritz Haber Center for Molecular Dynamics, Hebrew University of Jerusalem, Jerusalem 91904, Israel

Correspondence to:

E-mail: helden@fhi-berlin.mpg.de
Phone: +49 (0)30 8413 5615

Contents

- Excitation of a Two-level System in Helium Droplets …………………… S2
- Additional Experimental Spectra …………………………………………… S7
- Scaling Factor ……………………………………………………………….. S8
- Relative Energies at different levels of theory…………………………… S12
- Comparison of the experimental vs the harmonic calculated spectra at different levels of theory………………………………………..……… S13
- Effective mass of acidic hydrogens ……………………………………… S17
- Comparison of the experimental spectra of the deuterated species to the spectra calculated in the anharmonic approximation…………………… S19

Excitation of a Two-level System in Helium Droplets

The experiment described in the manuscript relies on the evaporation of helium atoms from a droplet after photon absorption. This evaporation is the consequence of vibrational energy, from the absorption of photons, being converted to phonons, leading to evaporation. In the following, we want to estimate the amount of energy that a single chromophore in a helium droplet during a light pulse can convert to phonons.

In a simplified picture, the excitation and relaxation processes can be described by rate equations for a two-level system, which include excitation, stimulated emission and relaxation of vibrational energy to phonons (Figure S1).

$$\frac{\mathrm{d}N_1}{\mathrm{d}t} = \sigma F(t)N_0(t) - \sigma F(t)N_1(t) - k_r N_1(t) \tag{1}$$

$$\frac{\mathrm{d}N_0}{\mathrm{d}t} = -\sigma F(t)N_0(t) + \sigma F(t)N_1(t) + k_r N_1(t) \tag{2}$$

where F is laser flux [photons/(cm^{2}s)], σ is the absorption cross-section [cm^2/molecule] and k_r is the rate constant for relaxation.

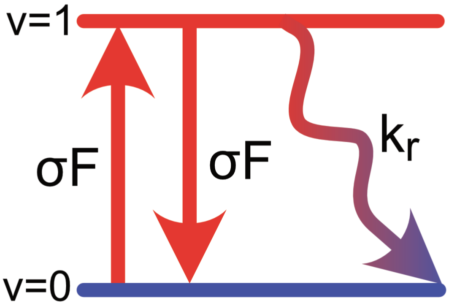

Figure S1. Excitation process in a two-level system.

We consider a single droplet with a single dopant molecule for which the sum of the populations is equal to one:

$$N_0 + N_1 = 1 \tag{3}$$

The number of photons of which the energy is transferred to phonons (leading to helium evaporation) ν_k during a light pulse with length T can be quantified by the integral of the product of the population of the higher energy level with the rate constant for relaxation over the duration of the light pulse:

$$\nu_k = \int_0^T k_r N_1(t) dt \tag{4}$$

The light from the FEL comes in macropulses of light that consist of micropulses. The macropulse length is 10 µs and the micropulse spacing is 1 ns. We assume that the micropulses have a gaussian temporal structure with a FWHM width of ~6 ps. Typically, quantum chemistry software reports band integrated IR intensities in km/mol. For the conversion to absorption cross sections in cm^2/molecule, we assume a width of the absorption band of 1 cm^{-1}. We further consider a typical intense absorption of 500 km/mol, a spot-size of 0.3 cm^2 and a photon energy of 1500 cm^{-1}.

The differential equations can then be integrated numerically with explicitly considering the time structure of the light. Figure S1 shows in the bottom part the first 10 ns of a macropulse with a total macropulse energy of 60 mJ and micopulse FWHM widths of ~6 ps. In the top two parts, the excited state population when using the parameters given in the text are shown for two relaxation rate constants.

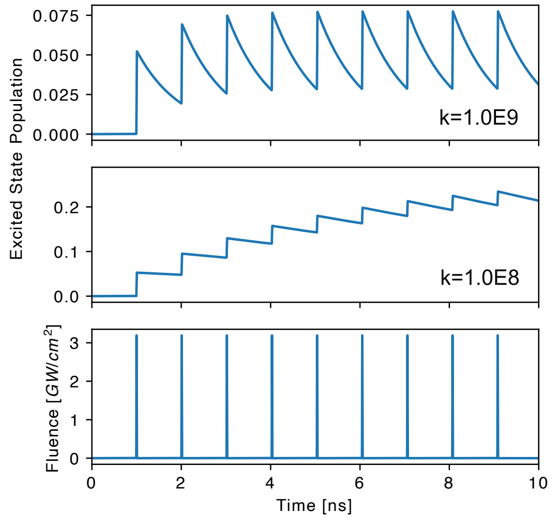

Figure S2. Bottom: the first 10 ns of a 10 µs FEL macropulse with total energy of 60 mJ and a micropulse FWHM width of ~6 ps. Middle and top: excited state populations for two different relaxation rate constants.

Equation 4 can then be used to numerically calculate the number of photons which relax after absorption and cause droplet heating.

For the case of a continuous-wave (cw) pulse, the equations above can be solved analytically. We consider a cw pulse with the same length as the macropulse (10 µs) and assume that the populations of the two energy levels, N_0 and N_1 reach equilibrium very fast, compared to the duration of the excitation pulse so that their time derivatives become zero.

$$\frac{\mathrm{d}N_0}{\mathrm{d}t} = \frac{\mathrm{d}N_1}{\mathrm{d}t} = 0 \tag{5}$$

Solving the equations yields the following expression for the excited state population:

$$N_1 = \frac{\sigma F}{2\sigma F + k_r}$$

(6)

When the flux of photons [photons/(cm^{2}s)] is constant over a pulse with length T, the number of photons transferred to phonon energy is given by:

$$\nu_k = k_r T \frac{\sigma F}{2\sigma F + k_r}$$

(7)

Figure S3 shows the number of photons converted to heat as a function of laser energy, obtained by numerically solving the equations 1-4 (solid lines) and from the approximation (eq. 7) for various relaxation rate constants. In polyatomic molecules, k_r is expected to be in the order of ps to ns[1]. The other parameters used are given above in the text above. It can be observed that the number of photons transferred to phonons is not only strongly dependent on the FEL macropulse energy, but also dependents strongly on the relaxation rate constant k_r.

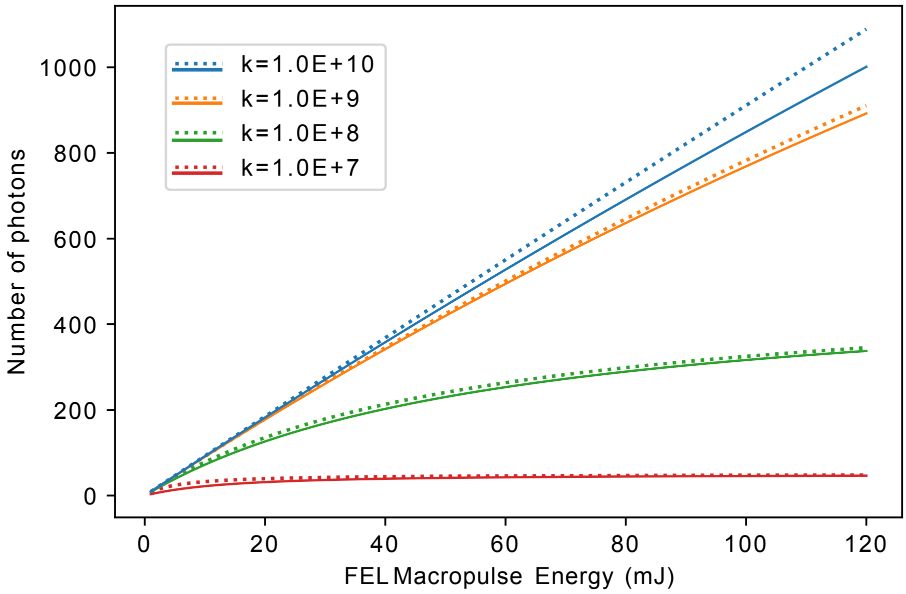

Figure S3. Number of photons converted to heat as a function of FEL macropulse energy for different constant rate constants. The solid lines are obtained from numerically solving eq. 1-4 and the dotted lines from the analytical model (eq. 7).

[1] Frank Madeja, Phineus Markwick, Martina Havenith, K Nauta, and RE Miller. Rotationally resolved infrared spectroscopy of h 2-and d 1-formic acid monomer in liquid he droplets. *The Journal of Chemical Physics*, 116(7):2870–2878, 2002.

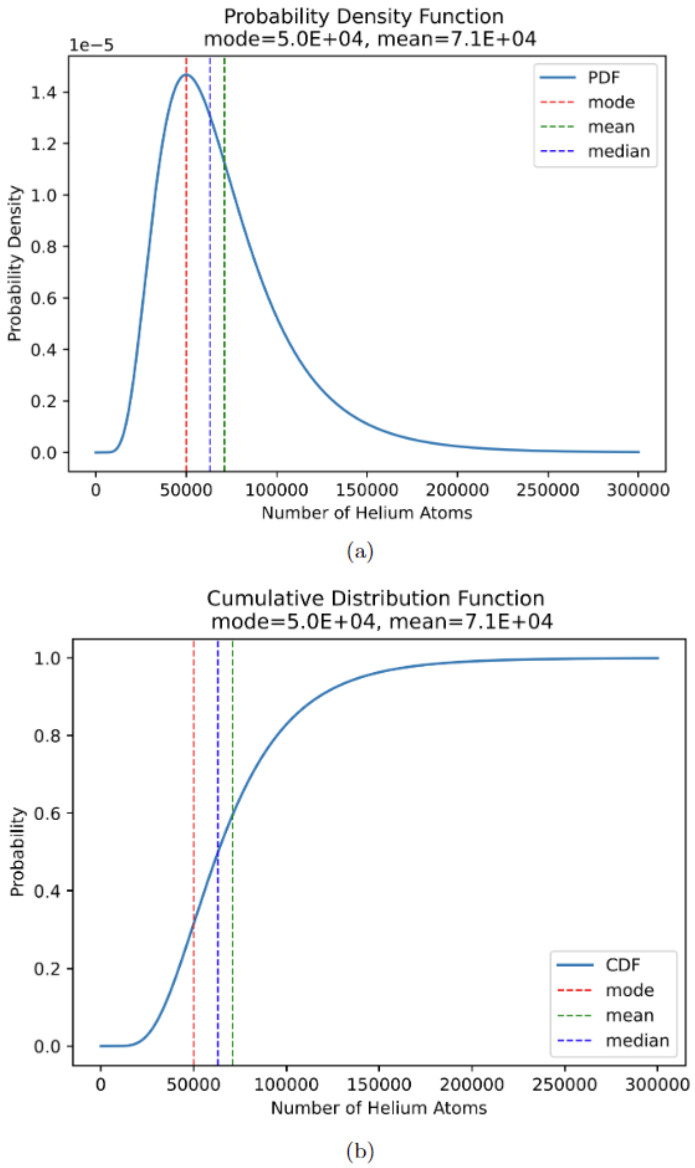

Figure S4. Probability Density Function (a) and Cumulative Distribution function (b) according to a log-normal distribution whit mode= 5.0E+04 and mean=7.1E+04, which reassemble the Droplet size-distribution of helium nanodroplets generated in the current experiment.

The size distribution of helium nanodroplets is anticipated to follow a log-normal distribution. Under the experimental conditions used in the presented experiment, the probability density function (PDF) is expected to have a mode (maximum) of 5.0E+04 and a mean of 7.1E+04 He atoms (Figure S4.a)[2]. By integrating the PDF over the range of droplet sizes, we obtain the cumulative distribution function (CDF) which provides insights into the likelihood of observing droplets of a certain size or smaller (Figure S4.b)

[2] Ana Isabel González Flórez. *Biomolecular Ions in Superfluid Helium Nanodroplets*. PhD thesis, Freie Universität Berlin, 2016

In our experiment, we observe the appearance of bare unsolvated ions after resonant IR absorption. Should this occur from the complete evaporation of the droplet, many photons have to be absorbed. Considering 5 cm^{-1} as binding energy for a helium atom to a droplet [3] we can relate the number of photons transferred to phonons to the fraction of doped droplets that are completely evaporated (Figure S5). The amount of unsolvated ions signal is strongly dependent on the relaxation rate constant k_r . Due to the droplet size distribution and the excitation dynamics, the observed signal depends non-linearly on and the absorption cross-section.

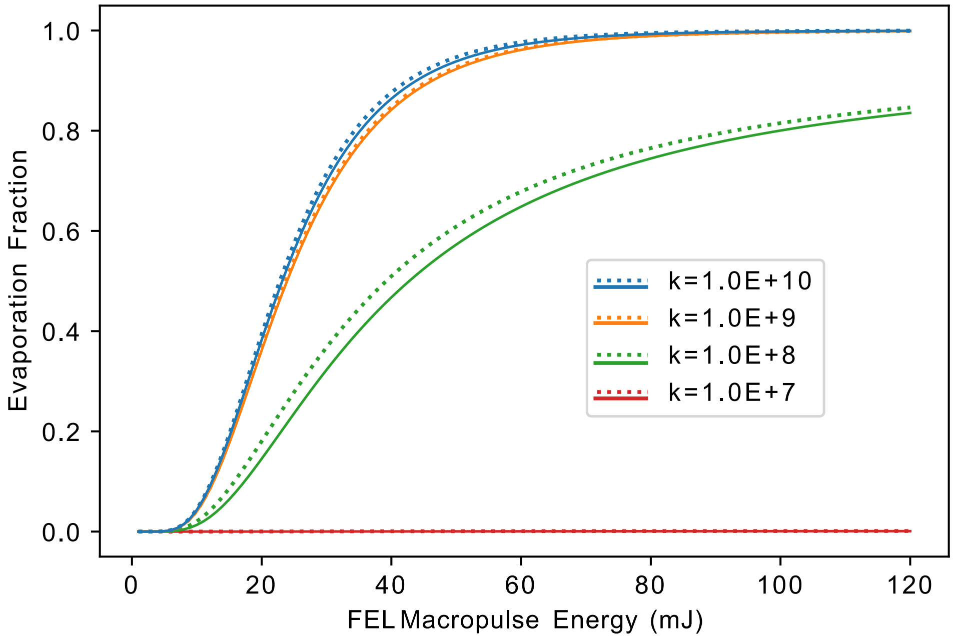

Figure S5. Evaporation fraction as a function of FEL macropulse energy considering the excitation of a 500 km/mol transition, a spot-size of 0.3 cm^2 and a photon energy of 1500 cm^{-1} for different relaxation rate constants. The helium nanodroplets are assumed to follow a log-normal droplet-size distribution with mode= 5.0E+04 and mean=7.1E+04. The solid lines represent the output of the model, which solves the differential equations numerically. The dotted line represents the output of the cw-approximation.

[3] J Peter Toennies and Andrey F Vilesov. Superfluid helium droplets: A uniquely cold nanomatrix for molecules and molecular complexes. *Angewandte Chemie International Edition*, 43(20):2622–2648, 2004.

Additional Experimental Spectra

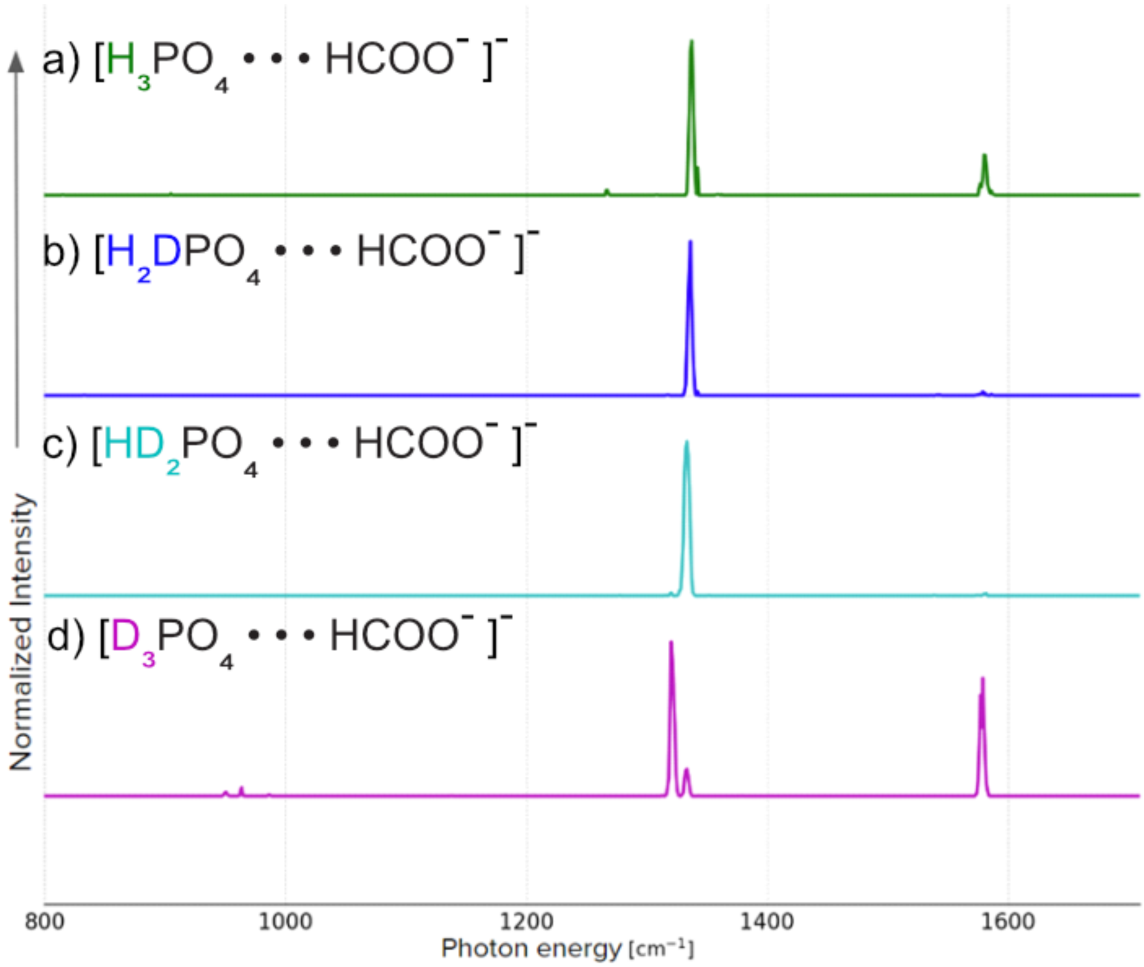

Figure S6. Experimental IR action spectra of the phosphoric acid-formate proton-bound dimer (a) [FP-H_3]$^-$ at different levels of deuteration (b) [FP-H_2D]$^-$, c) [FP-HD_2]$^-$, d) [FP-D_3]$^-$ measured at *very low* FEL macropulse energy (18 mJ).

Scaling factor

To select an adequate scaling factor for the harmonic vibrational frequencies, the ratio of the anharmonic (VPT2) to harmonic frequencies are calculated and averaged within the measured region. When performing this for all deuteration levels, a scaling factor of **0.975** is obtained. All calculations are performed at the B3LYP-D3(BJ)/Jul-cc-pV(T+d)Z level of theory.

$[FP\text{-}H_3]^-$

E(harm) (cm^{-1})	E(anharm) (cm^{-1})	Eanh/Eh	
3472.662	3323.996	0.95719	
3406.184	3112.102	0.913662	
2983.347	2591.223	0.868562	
2842.424	2622.546	0.922644	Average of the measured region
1626.259	1586.245	0.975395	0.972747
1403.203	1359.148	0.968604	
1386.556	1350.256	0.97382	
1360.184	1337.424	0.983267	
1294.518	1253.941	0.968655	
1232.704	1200.486	0.973864	
1161.439	1105.396	0.951747	
1067.643	1047.302	0.980948	
956.141	939.566	0.982665	
927.391	898.653	0.969012	
883.597	859.066	0.972237	
832.446	819.745	0.984743	
806.004	783.568	0.972164	
732.062	718.48	0.981447	
659.553	574.257	0.870676	
496.931	493.662	0.993422	
478.838	474.655	0.991264	
473.56	469.978	0.992436	
365.647	354.959	0.97077	
339.777	337.575	0.993519	
231.838	226.263	0.975953	
205.738	196.394	0.954583	
142.813	143.928	1.007807	
111.36	105.958	0.951491	
103.165	74.401	0.721185	
72.944	70.481	0.966234	
	Overall average:	0.956332	

[FP-H_2D]$^-$

E(harm) (cm^{-1})	E(anharm) (cm^{-1})	Eanh/Eh
3440.428	3098.503	0.900616
2986.605	2595.632	0.869091
2842.437	2625.808	0.923788
2501.567	2327.683	0.93049
1624.02	1581.807	0.974007
1395.887	1363.778	0.975997
1381.247	1348.301	0.975148
1358.127	1329.676	0.979051
1290.325	1254.019	0.971863
1204.881	1144.363	0.949773
1067.609	1047.741	0.98139
957.082	940.261	0.982425
939.588	915.231	0.974077
921.752	887.406	0.962738
843.03	815.025	0.966781
828.726	815.27	0.983763
781.337	764.806	0.978843
711.017	642.399	0.903493
521.951	473.181	0.906562
487.604	484.086	0.992785
474.972	470.853	0.991328
463.627	453.199	0.977508
359.629	348.796	0.969877
336.382	333.993	0.992898
231.463	226.309	0.977733
205.425	196.788	0.957955
142.521	144.371	1.012981
111.063	106.022	0.954611
101.601	76.467	0.752621
72.758	70.377	0.967275
	Overall average:	0.954649

Average of the measured region
0.972847

[FP-HD_2]$^-$

E(harm) (cm^{-1})	E(anharm) (cm^{-1})	Eanh/Eh
2990.729	2597.542	0.868531
2842.465	2621.079	0.922115
2523.281	2460.285	0.975034
2480.944	2327.445	0.938129
1622.09	1577.343	0.972414
1393.602	1361.157	0.976719
1375.486	1345.158	0.977951
1355.148	1323.48	0.976631
1287.414	1261.179	0.979622

Average of the measured region
0.976149204

1067.559	1047.491	0.981202
963.217	945.634	0.981746
940.212	917.141	0.975462
935.785	911.8	0.974369
917.311	881.179	0.960611
827.5	811.707	0.980915
804.129	779.123	0.968903
767.756	757.847	0.987094
568.803	573.966	1.009077
498.786	469.316	0.940917
480.048	475.407	0.990332
467.86	461.56	0.986534
452.582	436.721	0.964954
353.65	342.514	0.968511
333.659	331.624	0.993901
231.088	226.133	0.978558
205.112	196.789	0.959422
142.233	143.728	1.010511
110.668	105.601	0.954214
100.226	77.777	0.776016
72.565	70.16	0.966857
	Overall average:	0.963242

$[FP\text{-}D_3]^-$

E(harm) (cm^{-1})	E(anharm) (cm^{-1})	Eanh/Eh	
2845.799	2639.289	0.927433	
2528.454	2469.424	0.976654	
2480.942	2325.159	0.937208	
2185.64	2030.118	0.928844	Average of the measured region
1616.905	1577.526	0.975645	0.979412
1392.323	1360.54	0.977173	
1366.102	1342.831	0.982965	
1319.719	1301.47	0.986172	
1067.299	1048.229	0.982132	
998.91	978.337	0.979405	
961.749	942.246	0.979721	
938.571	916.011	0.975963	
913.259	890.248	0.974803	
826.167	812.695	0.983693	
807.286	782.689	0.969531	
763.491	754.761	0.988566	
669.522	646.717	0.965938	
568.031	574.171	1.010809	
493.029	465.972	0.945121	
475.376	468.86	0.986293	
461.202	457.896	0.992832	

452.569	436.756	0.965059
349.983	340.216	0.972093
331.577	329.329	0.99322
230.4	225.164	0.977274
204.667	196.955	0.962319
141.494	142.281	1.005562
109.842	105.103	0.956856
100.211	78.426	0.782609
72.565	70.311	0.968938
	Overall average:	0.967028

Relative Energies at different levels of theory

Table TS1. Comparison of relative energies of [FP-H_3^-] at different levels of theory without considering zero-point vibrational energy (ZPE).

Level of theory	B3LYP-D3(BJ)/Aug-cc-pV(T+d)Z	B3LYP-D3(BJ)/Jul-cc-pV(T+d)Z	WB97-XD/Jul-cc-pV(T+d)Z	MP2/Jul-cc-pV(T+d)Z
Structure	Relative Electronic Energy [kJ/mol] (EE)			
a)	0.0	0.0	0.0	0.0
b)	+8.8	+8.3	+9.8	+10.7
c)	+14.5	+13.9	+15.8	+16.6

Table TS2. Comparison of relative energies of [FP-H_3^-] at different levels of theory taking into account zero-point vibrational energy (ZPE)

Level of theory	B3LYP-D3(BJ)/Aug-cc-pV(T+d)Z	B3LYP-D3(BJ)/Jul-cc-pV(T+d)Z	WB97-XD/Jul-cc-pV(T+d)Z	MP2/Jul-cc-pV(T+d)Z
Structure	Relative Electronic Energy [kJ/mol] (EE + ZPE)			
a)	0.0	0.0	0.0	0.0
b)	+3.5	+3.1	+4.8	+5.8
c)	+9.6	+9.1	+11.2	+11.8

Comparison of the experimental spectra of FP-H_3 to the spectra calculated in the harmonic approximation at different levels of theory

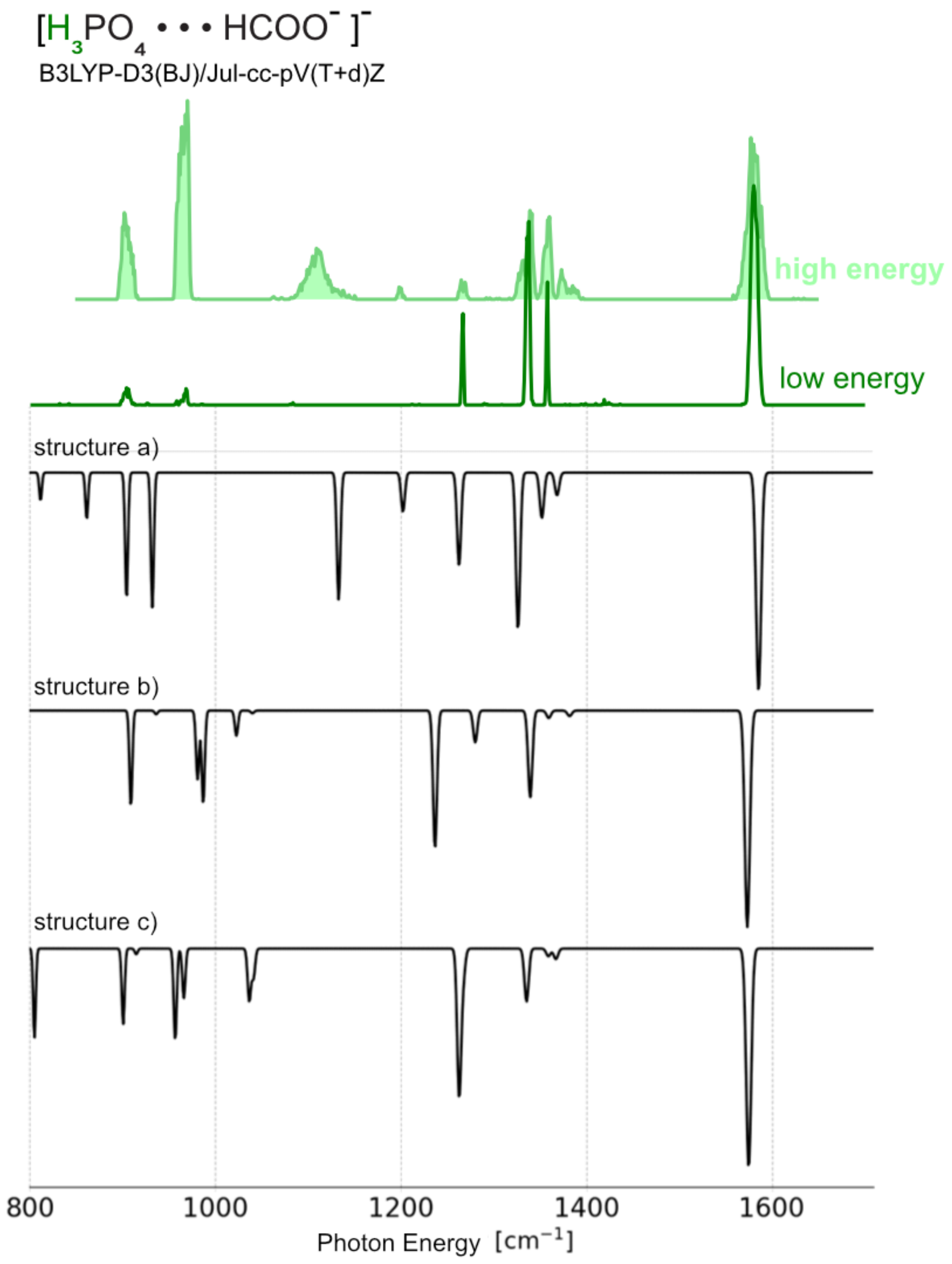

Figure S7. Experimental IR action spectra of the phosphoric acid-formate proton-bound dimer [FP-H_3]$^-$ at high (light green) and low (green) FEL macropulse energy in comparison with the calculated harmonic (0.975 scaled, black) IR spectra of the three lowest minima structures (a), b), c)) computed at the B3LYP-D3(BJ)/Jul-cc-pV(T+d)Z level of theory.

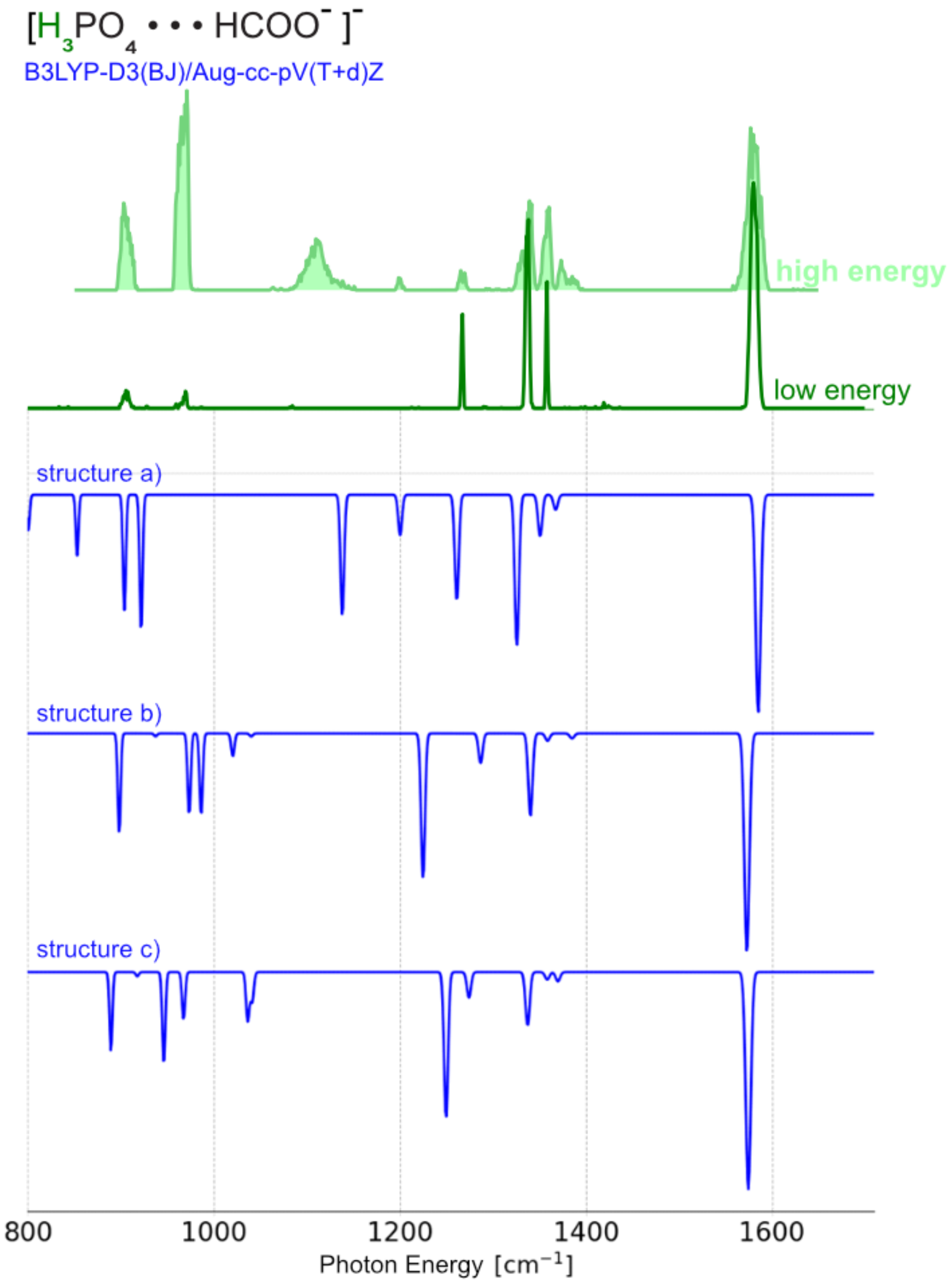

Figure S8 Experimental IR action spectrum of the phosphoric acid-formate proton-bound dimer [FP-H$_3$]$^-$ at high (light green) and low (green) FEL macropulse energy in comparison with the calculated harmonic (0.975 scaled, blue) IR spectra of the three lowest minima structures (a), b),c)) computed at the B3LYP-D3(BJ)/Aug-cc-pV(T+d)Z level of theory.

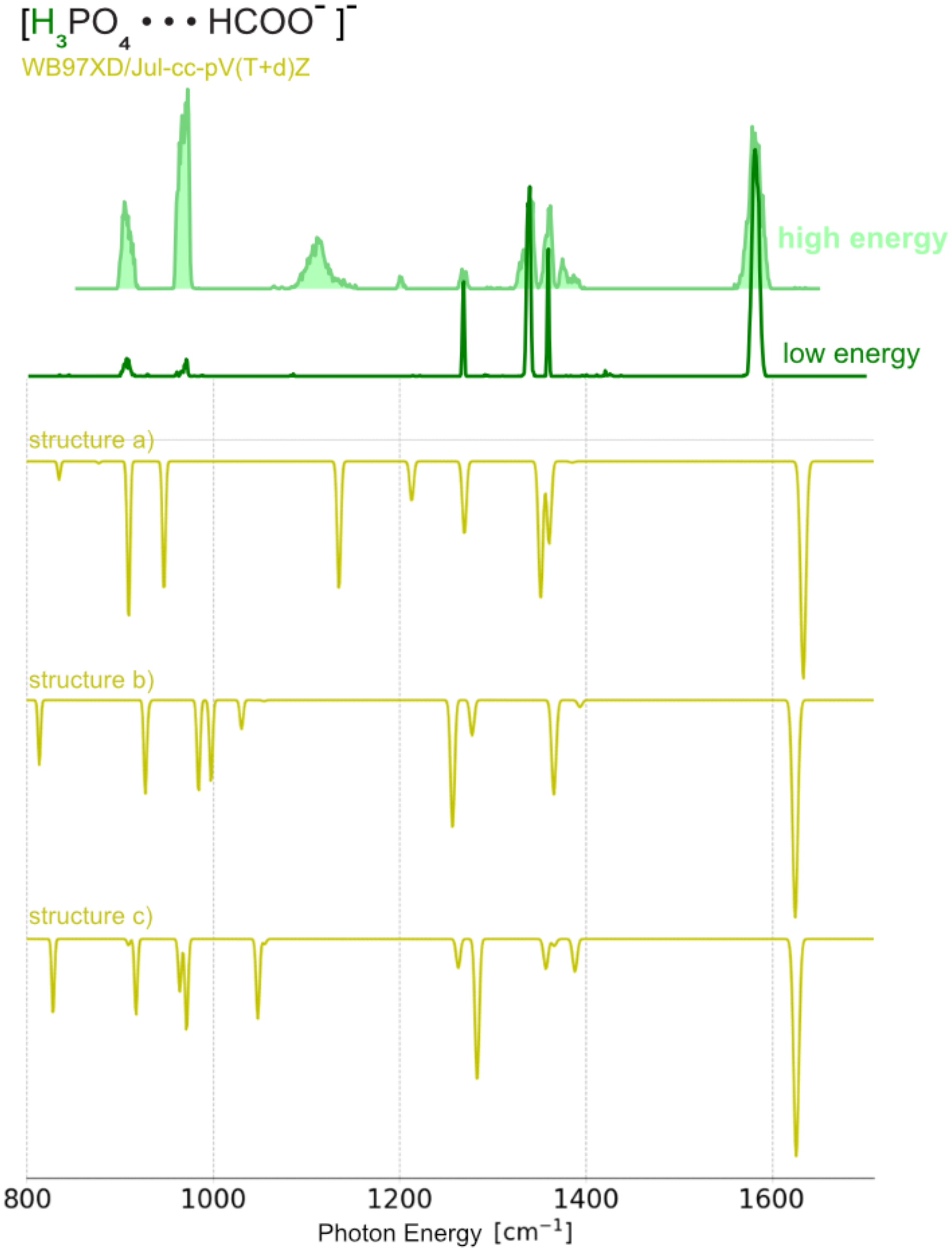

Figure S9 Experimental IR action spectra of the phosphoric acid-formate proton-bound dimer [FP-H$_3$]$^-$ at high (light green) and low (green) FEL macropulse energy in comparison with the calculated harmonic (0.975 scaled, yellow) IR spectra of the three lowest minima structures (a), b),c)) computed at the WB97-XD/Jul-cc-pV(T+d)Z level of theory.

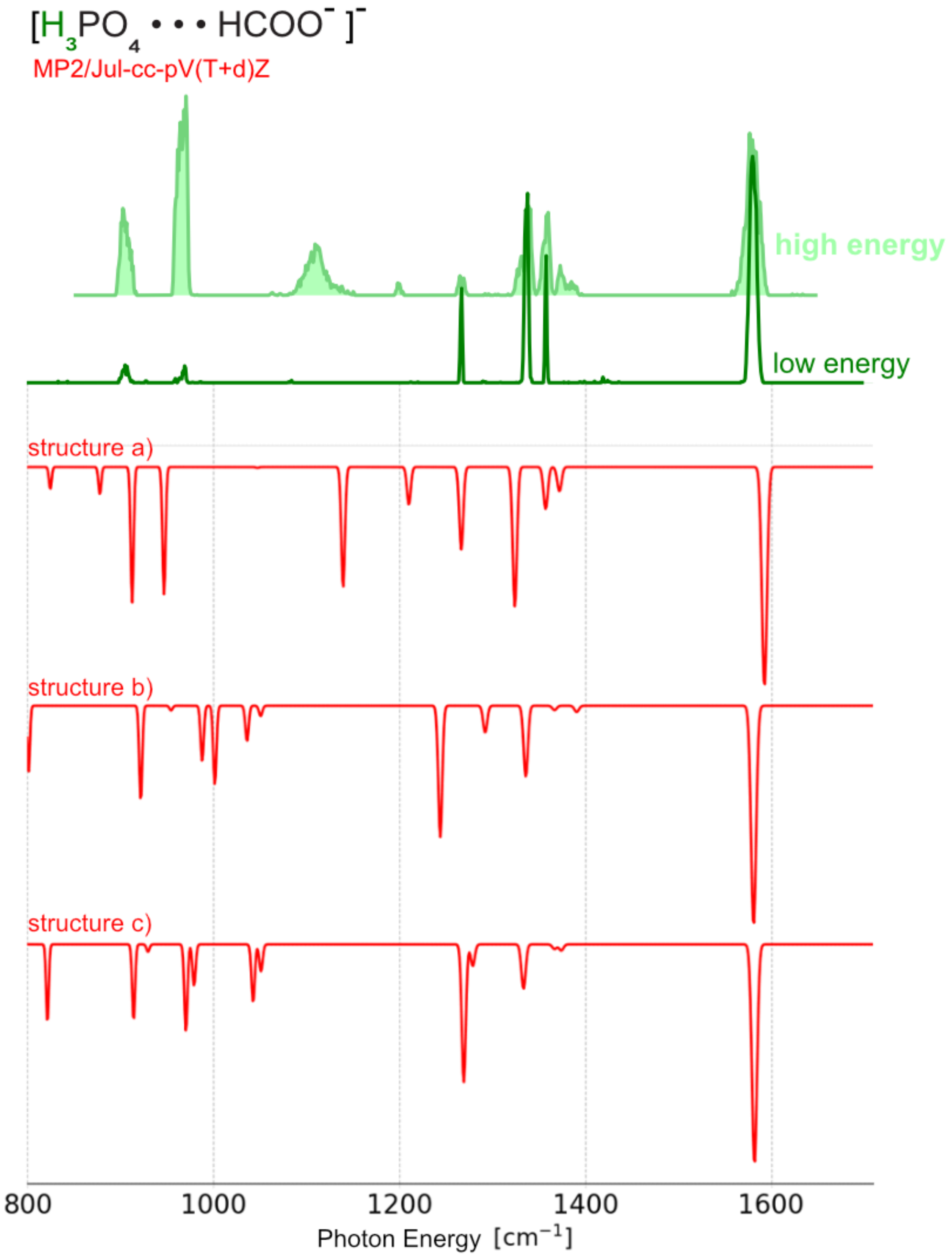

Figure S10 Experimental IR action spectra of the phosphoric acid-formate proton-bound dimer $[FP\text{-}H_3]^-$ at high (light green) and low (green) FEL macropulse energy in comparison with the calculated harmonic (0.975 scaled, red) IR spectra of the three lowest minima structures (a), b),c)) computed at the MP2/Jul-cc-pV(T+d)Z level of theory.

Effective mass of acidic hydrogens

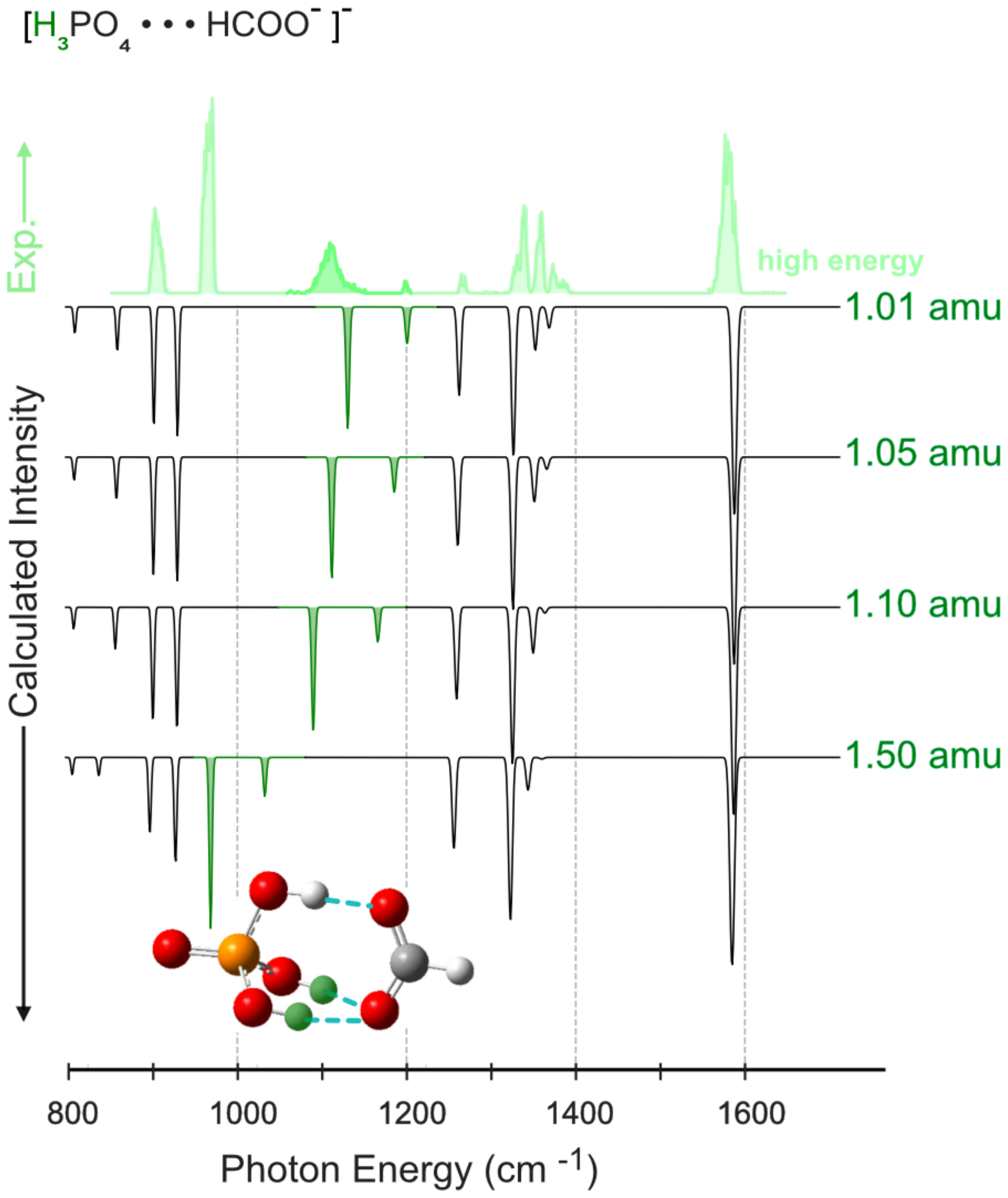

Figure S11 The theoretical vibrational bands calculated for $[FP\text{-}H_3]^-$ at the B3LYP-D3(BJ)/Jul-cc-pV(T+d)Z level of theory and scaled by a factor of 0.975 (black line). In the calculations, the mass of two hydrogen atoms (green highlight) is artificially increased by 1, 5, 10, and 50\%. The two bands that shift the most are highlighted in green. The calculations are compared to the high FEL macropulse energy experimental spectrum (top trace, light green filling).

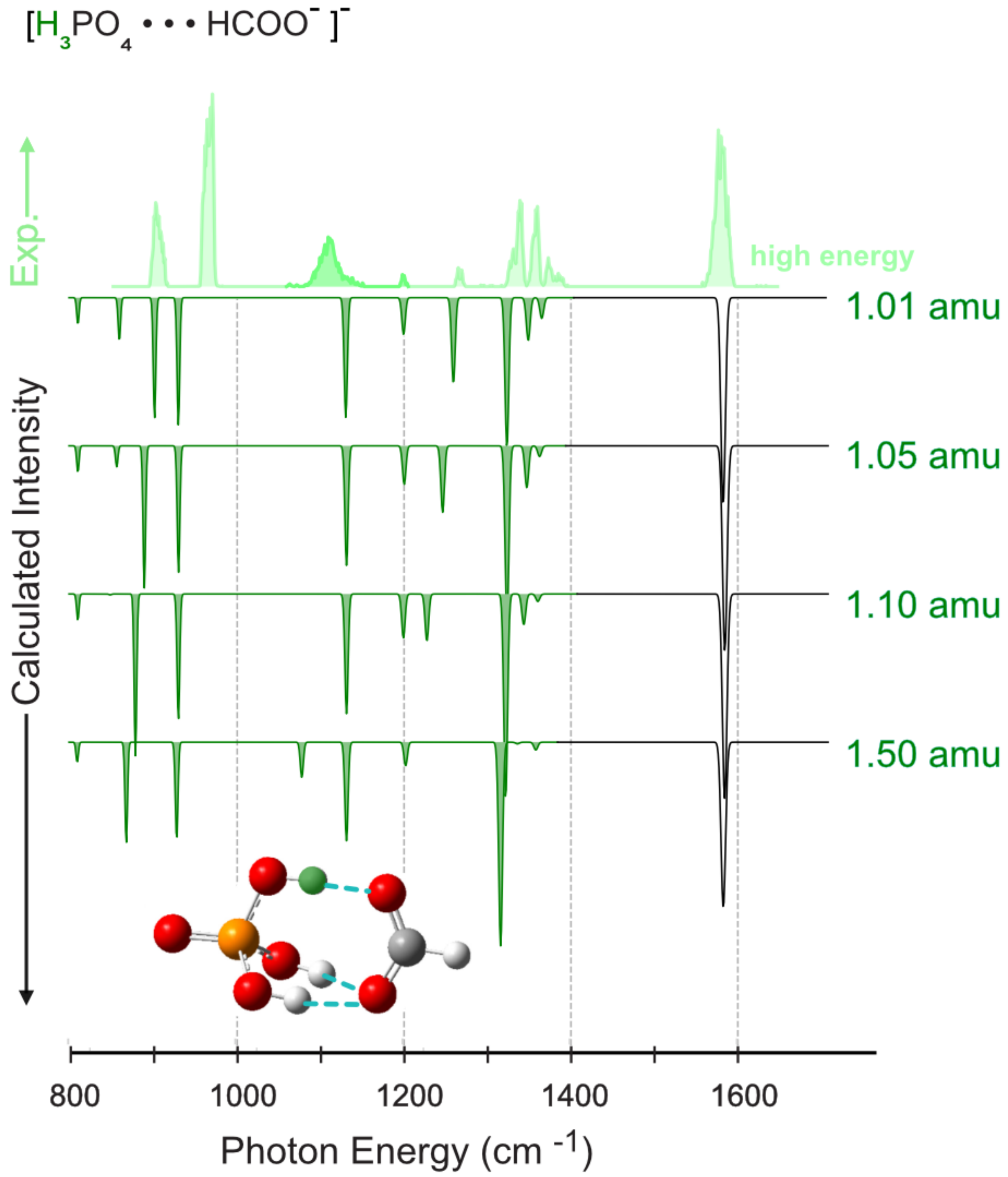

Figure S12 The theoretical vibrational bands calculated for $[FP\text{-}H_3]^-$ at the B3LYP-D3(BJ)/Jul-cc-pV(T+d)Z level of theory and scaled by a factor of 0.975 (black line). In the calculations, the mass of one hydrogen atom (green highlight) is artificially increased by 1, 5, 10, and 50\%. The bands that change the most are highlighted in green. The calculations are compared to the high FEL macropulse energy experimental spectrum (top trace, light green filling).

Comparison of the experimental spectra of the deuterated species to the spectra calculated in the anharmonic approximation

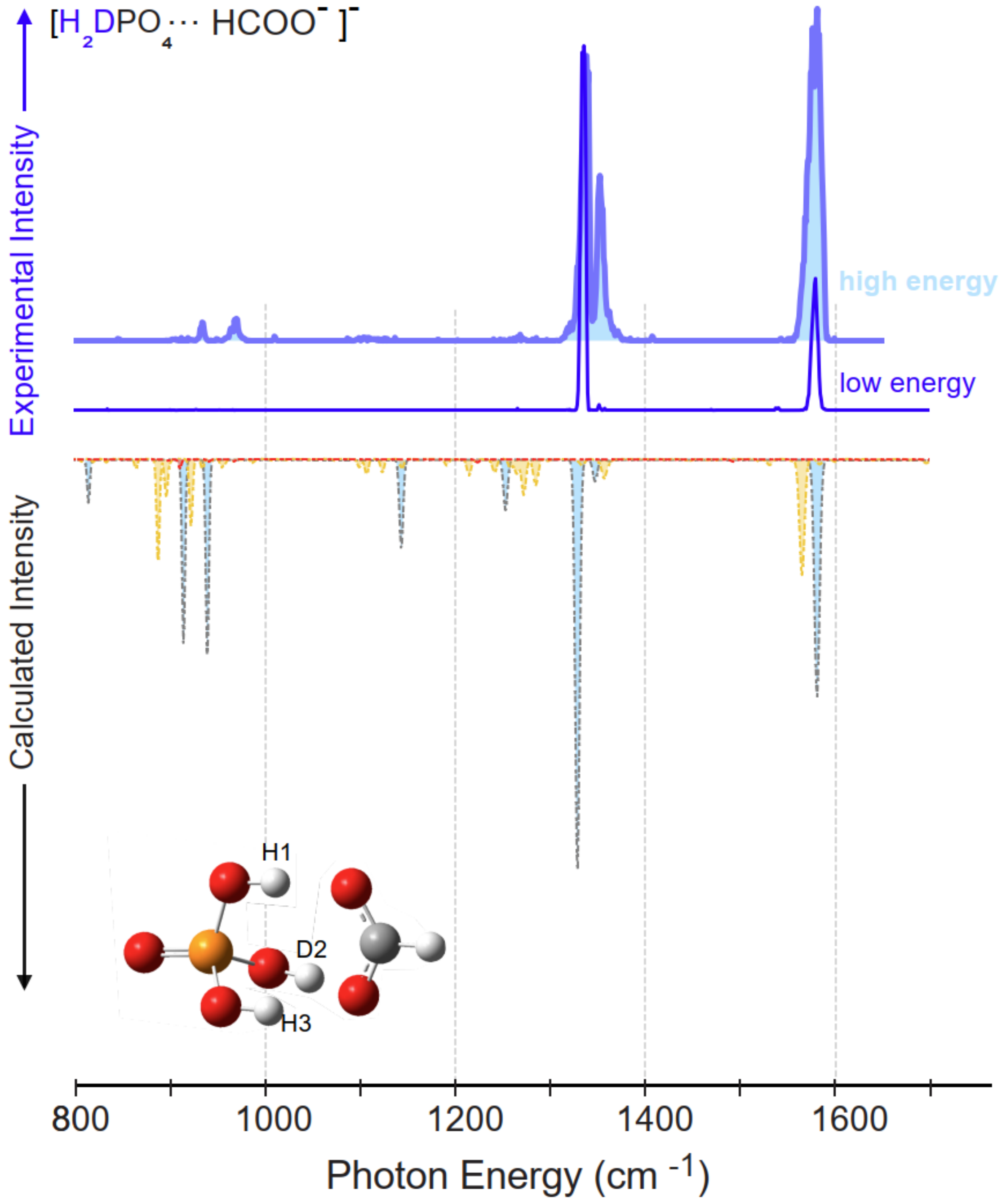

Figure S13 Experimental IR action spectra of the [FP-H_2D] $^-$ complex at high (filled light blue) and low (dark blue) FEL macropulse energy in comparison with the calculated IR spectra of structure a) computed at the B3LYP-D3(BJ)/Jul-cc-pV(T+d)Z level of theory. The calculated spectrum in the anharmonic approximation is shown in dashed lines. The spectrum comprises of bands stemming from fundamental modes (gray), combination bands (yellow), and overtones (red).

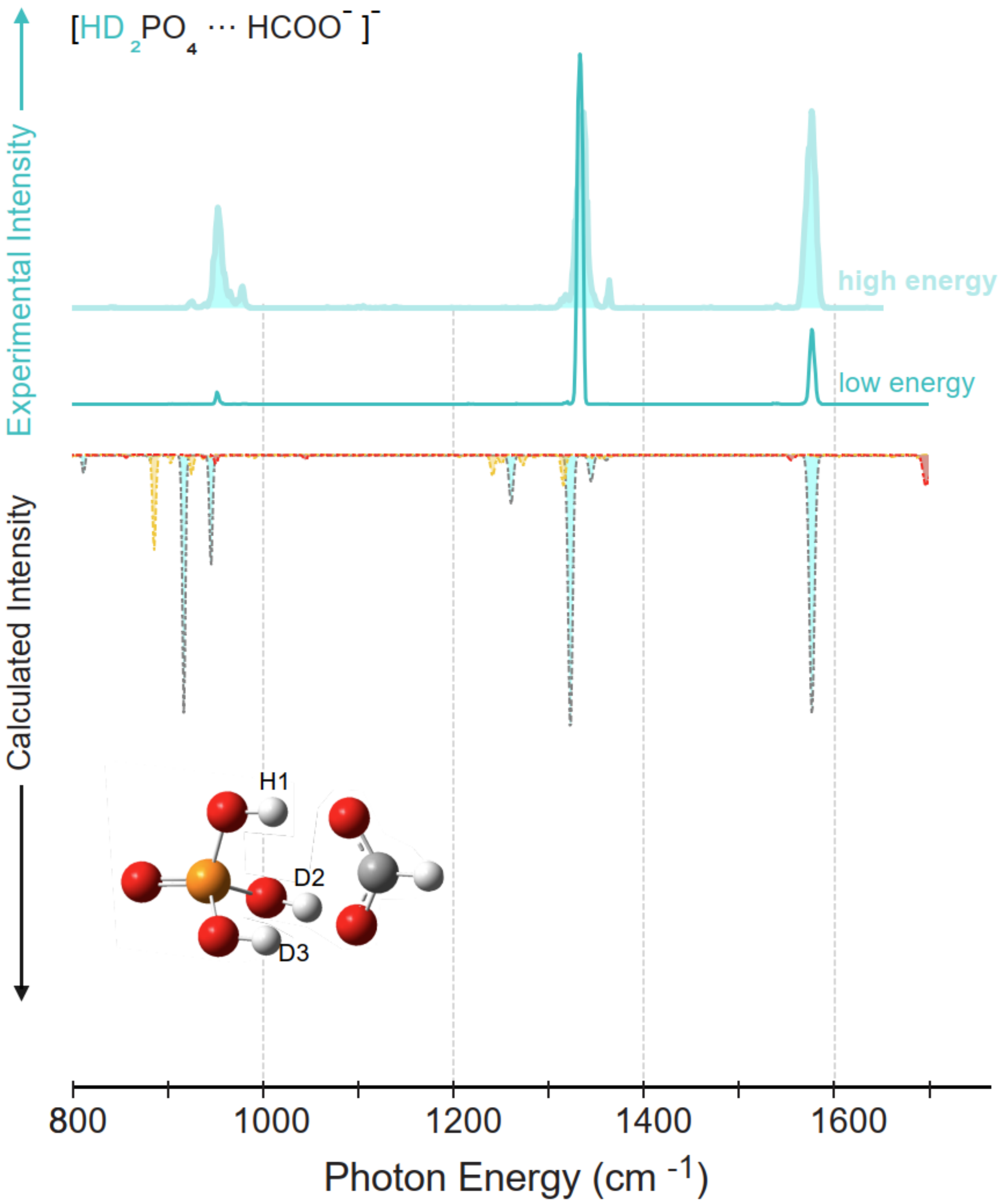

Figure S14 Experimental IR action spectra of the $[FP-H_2D]^-$ complex at high (filled light cyan) and low (dark cyan) FEL macropulse energy in comparison with the calculated IR spectra of structure a) computed at the B3LYP-D3(BJ)/Jul-cc-pV(T+d)Z level of theory. The calculated spectrum in the anharmonic approximation is shown in dashed lines. The spectrum comprises of bands stemming from fundamental modes (gray), combination bands (yellow), and overtones (red).

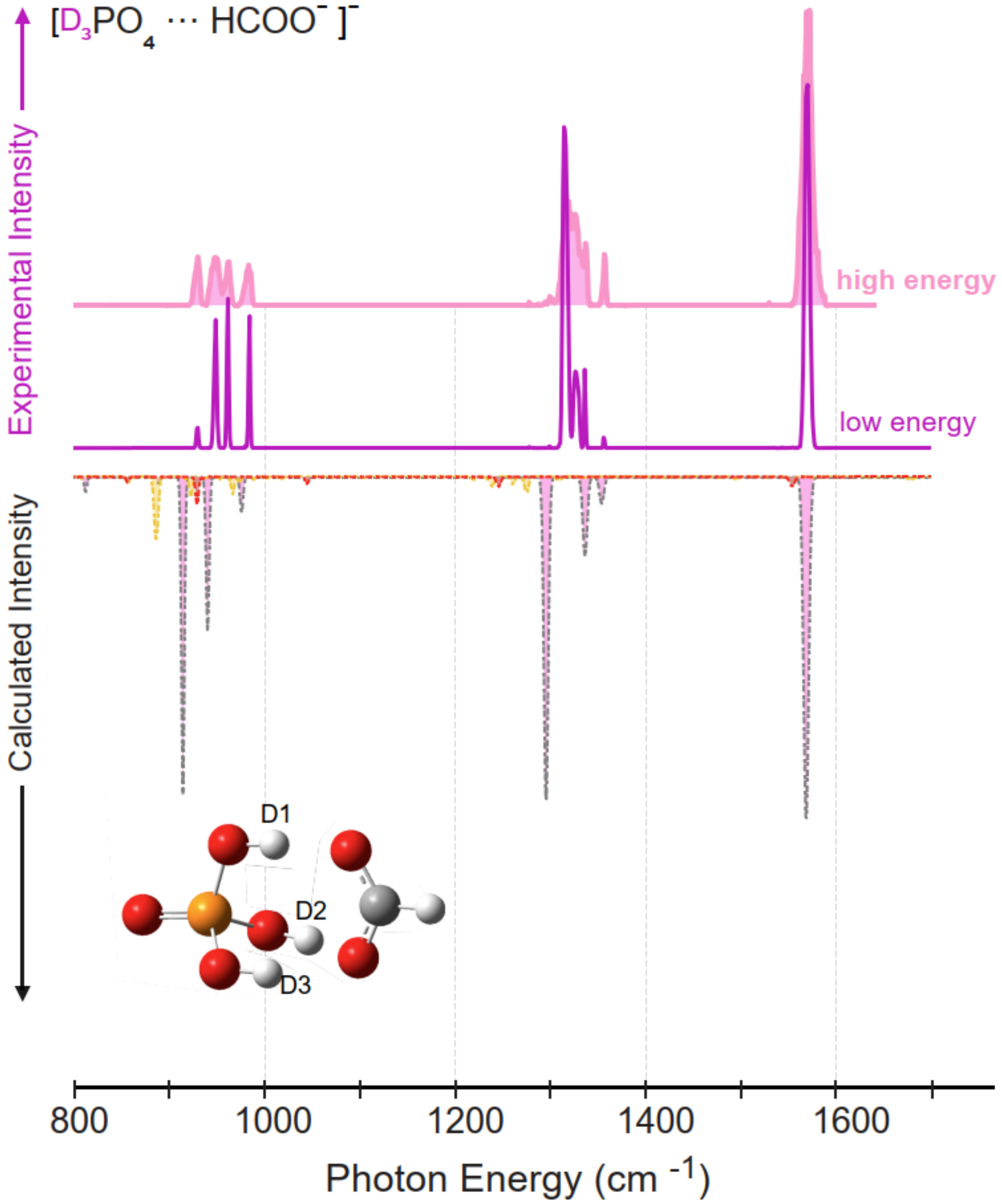

Figure S15 Experimental IR action spectra of the [FP-H_2D] $^-$ complex at high (filled light pink) and low (magenta) FEL macropulse energy in comparison with the calculated IR spectra of structure a) computed at the B3LYP-D3(BJ)/Jul-cc-pV(T+d)Z level of theory. The calculated spectrum in the anharmonic approximation is shown in dashed lines. The spectrum comprises of bands stemming from fundamental modes (gray), combination bands (yellow), and overtones (red).
